# Functional analysis of Rossmann-like domains reveals convergent evolution of topology and reaction pathways

**DOI:** 10.1371/journal.pcbi.1007569

**Published:** 2019-12-23

**Authors:** Kirill E. Medvedev, Lisa N. Kinch, R. Dustin Schaeffer, Nick V. Grishin

**Affiliations:** 1 Departments of Biophysics and Biochemistry, University of Texas Southwestern Medical Center, Dallas, Texas, United States of America; 2 Howard Hughes Medical Institute, University of Texas Southwestern Medical Center, Dallas, Texas, United States of America; Weizmann Institute of Science, ISRAEL

## Abstract

Rossmann folds are ancient, frequently diverged domains found in many biological reaction pathways where they have adapted for different functions. Consequently, discernment and classification of their homologous relations and function can be complicated. We define a minimal Rossmann-like structure motif (RLM) that corresponds for the common core of known Rossmann domains and use this motif to identify all RLM domains in the Protein Data Bank (PDB), thus finding they constitute about 20% of all known 3D structures. The Evolutionary Classification of protein structure Domains (ECOD) classifies RLM domains in a number of groups that lack evidence for homology (X-groups), which suggests that they could have evolved independently multiple times. Closely related, homologous RLM enzyme families can diverge to bind different ligands using similar binding sites and to catalyze different reactions. Conversely, non-homologous RLM domains can converge to catalyze the same reactions or to bind the same ligand with alternate binding modes. We discuss a special case of such convergent evolution that is relevant to the polypharmacology paradigm, wherein the same drug (methotrexate) binds to multiple non-homologous RLM drug targets with different topologies. Finally, assigning proteins with RLM domain to the Enzyme Commission classification suggest that RLM enzymes function mainly in metabolism (and comprise 38% of reference metabolic pathways) and are overrepresented in extant pathways that represent ancient biosynthetic routes such as nucleotide metabolism, energy metabolism, and metabolism of amino acids. In fact, RLM enzymes take part in five out of eight enzymatic reactions of the Wood-Ljungdahl metabolic pathway thought to be used by the last universal common ancestor (LUCA). The prevalence of RLM domains in this ancient metabolism might explain their wide distribution among enzymes.

## Author summary

Protein-ligand interactions are crucial to understanding molecular-level mechanisms of cell functions. A ligand-binding site frequently acts as a protein’s functional center, where its structural scaffold orients molecules in the correct conformation for proper function such as catalysis. These binding or active sites are usually conserved within protein families and can be used to define homologous proteins. However, even homologs may have diverged to functions requiring different active sites. Here we study the structural features and evolution of active sites of proteins containing the Rossmann-like motif (RLM). RLM proteins are numerous in nature and they constitute about 20% of all known 3D structures. Taking into account the prevalence of RLMs in proteins, analysis of their ligands provides general insights into enzyme evolution as well as proposes possible domain-based strategies for drug design in light of polypharmacology. Our analysis shows that RLM enzymes function predominantly in metabolism–these proteins cover 38% of reference metabolic pathways and are involved in numerous metabolic diseases. Being one of the most ancient folds, RLM enzymes could be traced to ancient metabolic pathways used by LUCA, which are still in use in some bacteria today.

## Introduction

The Rossmann-like fold [1, 2], being the most populated fold among α/β-topologies in the Protein Data Bank (PDB) [3], was described for the first time in a wide range of nucleotide-binding proteins that utilize diphosphate-containing cofactors such as NAD(H). The core of these protein structures included two sets of β-α-β-α-β units, forming a single parallel β-sheet (321456 topology) flanked by α-helices on either side [4]. A notable structural characteristic of this fold is a crossover between β-strands 3 and 4 that creates a natural cavity for binding nucleotides [5]. Rossmann-like folds, coined as “Rossmannoids”, occur in a large number of α/β three-layered sandwiches, many of which are thought to have evolved prior to LUCA from a primordial generic nucleotide-binding domain [6]. The extant set of Rossmann-like domains are linked to a large variety of metabolic enzymes and are capable of binding various ligands and small compounds necessary for their functions [7]. Given the prevalence and diversity of these domains, we theorize that comparative analysis derived from larger and more inclusive datasets will enable further functional and evolutionary insights into Rossmann-like domains. To this end we propose the broadest definition encompassing known Rossmannoids that is based on the smallest structural motif that can be enumerated. Such a definition should allow aggregation of many more Rossmannoid structures, bringing together some never before compared.

**Fig 1:**
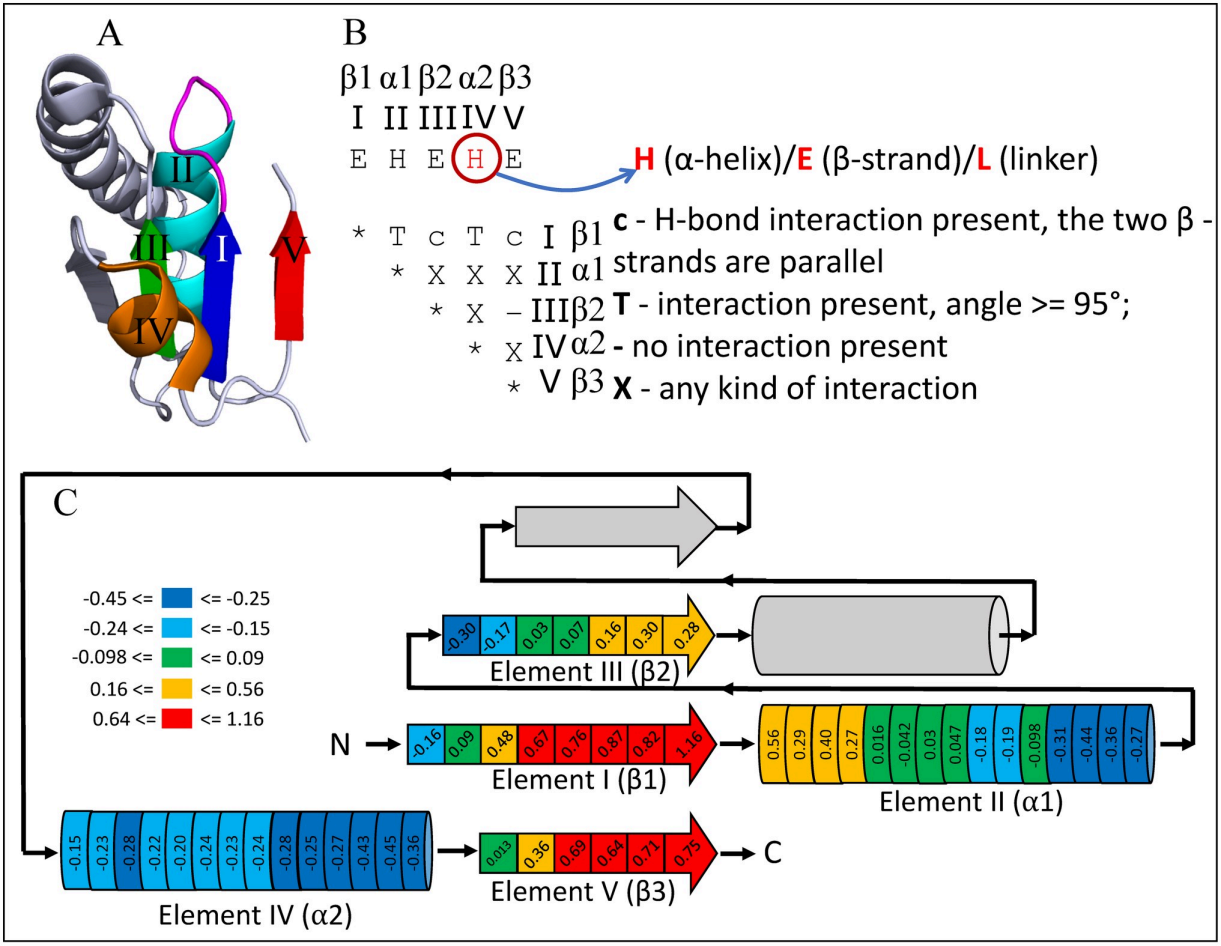
Minimal Rossmann-like motif (RLM) definition. (**A**) RLM SSEs adapted from 5-formly-3-hydroxy-2-methylpyridine 4-carboxylic acid (FHMPC) 5-dehydrogenase (PDB: 4OM8) are numbered and colored in rainbow, with magenta catalytic loop between first β-strand—element I (β1) and first α-helix—element II (α1). The second α-helix—element IV (α2) forms crossover between second β-strand—element III (β2) and third β-strand—element V (β3). The crossover loop is unstructured loop at the N-terminal part of α2. Element IV can be α-helix, β-strand, or loop. The unlabeled SSEs (colored in slate) are considered as an insertion to the RLM, which can occur between element III (β2) and element IV (α2) or in any of the loops connecting the RLM SSEs. (**B**) An interaction matrix defines RLM search strategy using ProSMoS program [22]. Interaction type “T” considers the angle between vectors corresponding to particular RLM elements. (**C**) RLM scheme with average AL2CO positional conservation index [23] among family level representatives. RLM bins are colored according to conservation index from blue (not conserved) to red (highly conserved), non-RLM elements are shown in gray. Left side of all SSE corresponds to N-terminus, right side to the C-terminus.

A Rossmann-like structure is frequently described as a doubly-wound, three-layer α/β/α sandwich with a central parallel β-sheet. Proteins containing our target minimal Rossmann-like motif (RLM) should maintain the "doubly-wound α/β/α sandwiches," topology (Fig 1). The central β sheet should contain at least two parallel β-strands and at least one α-helix on either side of the β sheet. The α-helix on one side connects the first two parallel strands, forming the first “wind”. The α-helix on the other side should connect this β-α-β unit with the last β-strand, which goes back to form hydrogen bonds with the first β-strand, giving the motif its second "wind". As a side note, we found that in some homologs this second α-helix can be replaced with a three-stranded β-sheet, so we further relaxed the requirement for this secondary structure element (see "Definition" below). RLM domain consists of two halves, which interface forms a cavity for ligands binding, right between the first β-strand of the protein and the first β-strand after the crossover. Notably, the major functional site of the ancient nucleotide-binding domain reside within this minimal definition: the N-terminal turn of the first α-helix (α1, see Fig 1A and 1B for the RLM SSE numeration) frequently binds phosphates, and the crevice between the first and the third β-strands β1 and β3 formed by the crossover accommodates larger substrates or cofactors, that are formed by the most conserved residues (Fig 1C).

The binding of ligands to proteins plays a major role in the regulation of most biological processes such as signal transduction and cellular metabolism [8]. Analysis of protein-ligand interactions is crucial, not only for understanding the regulation of biological functions, but also for drug design. Several databases are useful in identifying and classifying protein ligands. Frist, the Enzyme Commission number (EC number) classification scheme for enzyme-catalyzed reactions provides a valuable resource for functional classification of proteins, including their ligands [9] and metabolic pathways [10]. The hierarchical four-digit EC numbering system reveals the principal enzyme class at the broadest level (first digit) to specific substrate at the last level (fourth digit). Second, the Kyoto Encyclopedia of Genes and Genomes (KEGG) database [10] outlines the substrates, products and cofactors for each enzyme, providing a way to define biologically relevant ligands in RLM structures. Third, the ClassyFire database [11] constructs a chemical taxonomy along with a fully annotated chemical ontology. The hierarchical organization of chemical taxonomy for most compounds includes four major levels: kingdom, superclass, class and subclass. In the current work, we use the first three major levels of this database to classify ligands. For example, arginine belongs to “Organic compounds” kingdom, “Organic acids and derivatives” superclass, and “Carboxylic acids and derivatives” class.

Over decades, drug discovery studies have focused on identifying selective drugs to target a single mechanism, acting as unique ligands or “keys” for each specific molecule or “lock” [12]. However, drug design at the single molecular target level does not consider other affected processes. Recently, the validity of the lock-and-key model has been questioned, partly because of off-target effects [13]. Recently, effective drugs have been recognized as binding multiple cellular targets [14]. Now, a new drug discovery paradigm dubbed “polypharmacology” (i.e. that clinical effects are often because of the interaction of single or multiple drugs with multiple targets) is emerging [12]. Taking into account the prevalence of Rossmann-like folds in the world of proteins, analysis of their ligands provides an understanding of enzyme evolution as well as proposes possible targets for drug design in light of polypharmacology.

We primarily rely on Evolutionary Classification of protein Domains (ECOD) database [15] for our study on RLM domains. ECOD is an evolutionary-based classification of domains into a hierarchy that consists of five levels: architecture (A), possible homology (X), homology (H), topology (T), and family (F) [16]. The pilot version of ECOD was partially derived using SCOP as a starting point [17]. ECOD differs in its hierarchal organization from SCOP in several key details that have been described elsewhere [15, 18]. Briefly, ECOD recognizes more distant homology than SCOP and it prioritizes evolutionary relationships over folds, resulting in T-groups that distinguish homologs of different topology, whereas both ECOD and SCOP focus on homology and relies on sequence similarity, another recognized classification scheme, CATH, [19] emphasizes structural similarity. Finally, while ECOD is constantly updated with new structures released in PDB, SCOP and CATH are not as complete in their PDB coverage. Nevertheless, these alternative approaches lead to domain boundary definitions which are similar for most structurally characterized proteins. However, ECOD tends to partition proteins into slightly longer domains than CATH and slightly shorter domains than SCOP. The ECOD homology group level includes domains with very distant homologous relationship. This level corresponds to CATH homologous superfamily level, which groups together somewhat distant domains, and SCOP superfamily level, which groups together more similar domains [15] (See S1 Table for comparison of RLM-containing domains classification in ECOD, SCOP and CATH).

In this work, we identify all RLM domains in the protein structures classified in ECOD and assign their functional ligands using EC reactions [9], KEGG compounds [20] and UniProt KB cofactors [21]. RLM enzymes (i.e. that have EC numbers) function mainly in metabolism, with the top three over-represented pathways being nucleotide, energy, and amino acid metabolisms. By combining the evolutionary information from ECOD with these functional assignments, we find examples of both convergent and divergent evolution of RLM enzyme reactions and ligand binding sites. By analyzing ligands in PDB structures of RLM domains, we identify 20 superclasses of organic and inorganic compounds that bind to RLM enzymes. Of these classes, 15 are likely relevant to the protein function, with “Nucleotides and analogs” being the most populated superclass. We found that strong inhibiting agents, such as methotrexate, can inhibit multiple non-homologous RLM enzymes. We suggest that the presence of multiple methotrexate-binding sites among multiple non-homologous RLMs is particularly relevant in the light of polypharmacology theory.

## Results and discussion

### Definition, identification and evolutionary classification of RLM domains

A minimal RLM folding unit is defined as a three-layer α/β/α sandwich, with at least three parallel β-strands and a crossover between the second (element III corresponds to β2, Fig 1) and third β-strands (element V corresponds to β3, Fig 1). We refer to the secondary structure elements (SSEs) that comprise the RLM using “β” for β-strands and “α” for α-helices, together with the arabic numeral of the motif element outlined in Fig 1. For example, element I represents the first β-strand and is designated “β1”. Using this RLM definition, more than 80,000 (36,000 non-redundant by sequence) RLM domains were detected in the ECOD database. These domains were found in 28,489 (~20% of all known protein structures) PDB structures.

RLM domains belong to 1259 ECOD protein family groups (F-groups) that can be further grouped into 202 topology (T-groups) and 163 homology groups (H-groups) whose members have curated evolutionary relationships by experts based mainly on sequence and structure similarity [16]. These H-groups are further classified into 26 groups of possible homology (X-groups) (S1 Fig). X-groups require domains to have similarity in architecture and topology, and perhaps some functional similarity to suggest that homology between these structures is, in principle, possible. However, convincing evidence for homology of domains within a single X-group is presently lacking. Additional protein structures determined in the future, as well as improved homology inference tools, will allow us to assemble definitive evidence of homology and either merge some of these proteins into a single H-group or place them into different X-groups. Thus, X-groups represent a gray zone in our classification. On the one hand, we suspect that these domains may be homologous, on the other hand, we acknowledge that confident decision is not possible with the data and tools we have at our disposal today. Furthermore, it is frequently easier to argue for homology than against it. A homology link between proteins (by sequence, structure, and frequently function), supported by multiple lines of evidence, can bring proteins in an H-groups, but in many cases, it is unfeasible to fully exclude the possibility that different X-groups are necessarily analogous. In some instances homology at the level of the whole domain (i.e. some gene duplication of the whole RLM structural core) seems unlikely, although some repetitive elements within each structure, such as a single β/α unit, could be homologous and might have changed beyond our ability to detect them. As such, the ECOD classification of RLM domains reveals numerous instances of potential analogy and establishes a range from as few as several to as many as 163 (from the number of different H-groups) cases of convergent evolution which adopt the same SSE motif within various structure frameworks.

In our data set, we observed proteins with RLM SSEs that cannot be linked to other RLM domains using sequence or structure similarity. These distinct domains might not have originated from a common Rossmann-like ancestor. In some cases, the RLM does not belong to the conserved structural core and is instead acquired as recent decoration. We define these cases as proteins that contain a minimal Rossmann-like equivalent (RLE). Only a single H-group contains an RLE: TIM barrel homology group (ECOD: 2002.1), where the RLE is formed by insertion to the conserved core of the TIM-barrel domain and, being the basis of lid domain movement, is a functionally important structural feature of this family (S1 Appendix; S2 Fig).

According to the ECOD classification nearly all X-groups in our dataset belong to “α/β three-layered sandwiches” architecture group (A-group), with three exceptions: the TIM barrel proteins containing an RLE belong to “α/β barrels”, the STIV B116-like viral proteins (ECOD: 4259.1), containing unique domains from archaeal viruses, belong to “α+β complex topology” (discussed in our recent paper [24]). Finally, the OmpH-like proteins (ECOD: 5094.1) function as obligate trimers and belong to “α+β duplicates or obligate multimers”. Also, not all members of “α/β three-layered sandwiches” A-group are Rossmann-folds: 26 X-groups do not contain a RLM. There are couple of reasons for this: these proteins may not have crossover and may have antiparallel β-strands at the RLM location.

The ECOD H-group level corresponds to CATH homologous superfamily level and SCOP superfamily level. We attempted to assign RLM domains to CATH and SCOP using representative RLM domains from each F-group. Since CATH and SCOP are not as complete as ECOD, about 49% and 84% of RLM domains can be mapped to SCOP’s and CATH’s classifications, respectively (S1 Table). CATH and SCOP are more conserved in recognizing remote homology than ECOD, and they tend to classify the same set of RLM domains that are shared among all three classification schemes into many more (1.35 times more for SCOP and 1.48 times more for CATH) superfamilies than ECOD. CATH, with the emphasis on structure instead of homology, indeed recognize the similarity between RLM domains and classify 72% of them in “Rossmann fold” (T: 3.40.50), whereas in SCOP, RLM domains are scattered among many different folds in the alpha/beta proteins class.

We divided each RLM element into bins and analyzed average positional conservation index [23] among family level representatives (see Materials and methods, Fig 1C, S3 and S4 Figs). Average conservation index values for each bin are shown in Fig 1C and colored from blue (not conserved) to red (highly conserved). Results of this analysis revealed that bins with the highest conservation index located at the C-terminal end of β1, N-terminal end of α1 and C-terminal end of β3. The most common RLM binding sites are located in these areas and are described below throughout the Results section. Calculation of average conservation index inside RLM (0.116±0.008) versus outside of RLM (-0.276±0.004) for family level representative domains revealed that the conservation inside RLM is significantly higher than outside of RLM according to Kolmogorov-Smirnov test (P-value < 0.0001) [25]. For the majority of RLM proteins binding site locates inside RLM, however there are exceptions, which are also discussed below.

Several groups hypothesized that complex protein folds may have arisen from short peptides through multiple fusion and duplication events [26–29]. It follows that some modern protein folds arose from numerous duplications of common short peptide ancestors but are sufficiently ancient that their shared evolutionary history cannot be discerned. Alternatively, other modern protein folds may be polyphyletic in origin; evolved from the fusion of multiple, distinct peptide ancestors (which are themselves the products of far earlier divergences). Studying the ligand-binding features of diverse groups such as RLM proteins could help to reveal the nature of functional constraints on fold diversification as well as to suggest strategies for developing potential drug targets for diseases that are associated with these proteins.

### Summaries of results section content

To ease the navigation through the subsections, we divide them into two groups: general principles of ligands binding by RLM proteins and specific examples. We summarize the general ideas in each subsection below.

General principles of binding ligands by RLM proteins include the following subsections:

1. RLM-containing proteins play a major role in metabolic pathways: 70% of RLM-containing proteins take part in global metabolism and other major KEGG pathways. Reactions catalyzed by these proteins are significantly overrepresented in nucleotide, energy and amino acids metabolism.
2. RLM homologs can catalyze diverse reaction types: distribution of reaction types in the largest RLM F-groups. The rhodanese family catalyzes diverse reactions using similar active site chemistry.
3. Converging to similar functions: Non-homologous RLM families can catalyze the same reactions: discussion of distant RLM enzymes topologies that catalyze the same reactions.
4. RLM enzymes bind a diverse set of ligands: discussion of the superclasses of major ligands and their binding modes for the top 10 most populated RLM H-groups.
5. Diverse binding modes for inorganic cofactors in RLM enzymes: for the most common metal cofactor Mg^2+^, RLM domains appear to have evolved multiple different structural contexts (41 ECOD H-groups) and catalytic activities (205 EC numbers).

Specific examples include the following subsections:

1. Evolution of nucleotide/nucleoside ligand binding in RLM enzymes: binding modes reflect homology groups. RLM enzymes from different H-groups reveal different binding modes for the same ligand.
2. Divergent evolution of nucleotides/nucleosides binding sites: several cases of homologous RLM enzymes reveal divergent evolution of binding sites to accommodate different ligands.
3. Allosteric nucleotides/nucleosides binding sites in RLM domains: examples of RLM allosteric binding sites, which could be prospective targets for drugs
4. Iron-sulfur cluster reactions: the most common Fe-S binding site in our dataset binds [4Fe-4S], which functions mainly in oxidoreductase reactions. RLM enzymes take part in ancient Wood-Ljungdahl (WL) or reductive acetyl-coenzyme A pathway.
5. RLM domains bind nucleic acids: the second largest class of pathways, which includes 21 RLM enzymes, is “Genetic Information Processing”. These 21 enzymes can only be observed in a single pathway–“Aminoacyl-tRNA biosynthesis” and they bind RNA.
6. RLM enzymes inhibited by methotrexate reveal its side effects significance: methotrexate is capable to inhibit RLM enzymes from different H-groups, which might cause severe side effects.

### RLM containing proteins play a major role in metabolic pathways

To understand the diversity of chemical reactions catalyzed by RLM domains, we assigned EC numbers to all RLM structures using PDB [3] and UniProt KB [21] annotations. About two-thirds of the RLM PDB structures (18,824 out of 28,489) were assigned to 1472 EC numbers, leaving 9,665 structures unassigned and suggesting that a majority of RLM structures are enzymes (statistics in S4 Table: http://prodata.swmed.edu/rossmann_fold/lig_master_table/).

**Table 1:** Top three ECOD Family groups with largest number of unique EC numbers.

F-group name	F-group ID	Number of unique EC
enoyl-(Acyl carrier protein) reductase	2003.1.1.417	51
aminotransferase class V	2111.77.1.72	46
alpha/beta hydrolase	2111.78.1.7	40

We further map these enzymes to metabolic pathways in KEGG. While 30% (421 out of 1378 EC numbers without dashes) of assigned RLM EC numbers do not map to any KEGG pathways, the remaining 70% (957 out of 1378) take part in global metabolism (939 EC numbers) and other major KEGG pathways, such as genetic information processing (21 EC numbers) and environmental information processing (9 EC numbers). EC numbers of RLM proteins (indicated by black arrows in S7 Fig, http://prodata.swmed.edu/rossmann_fold/lig_online_fig1/) belong to numerous and diverse modules in the KEGG reference metabolic pathway map, including reactions from all major KEGG pathway categories.

**Fig 2:**
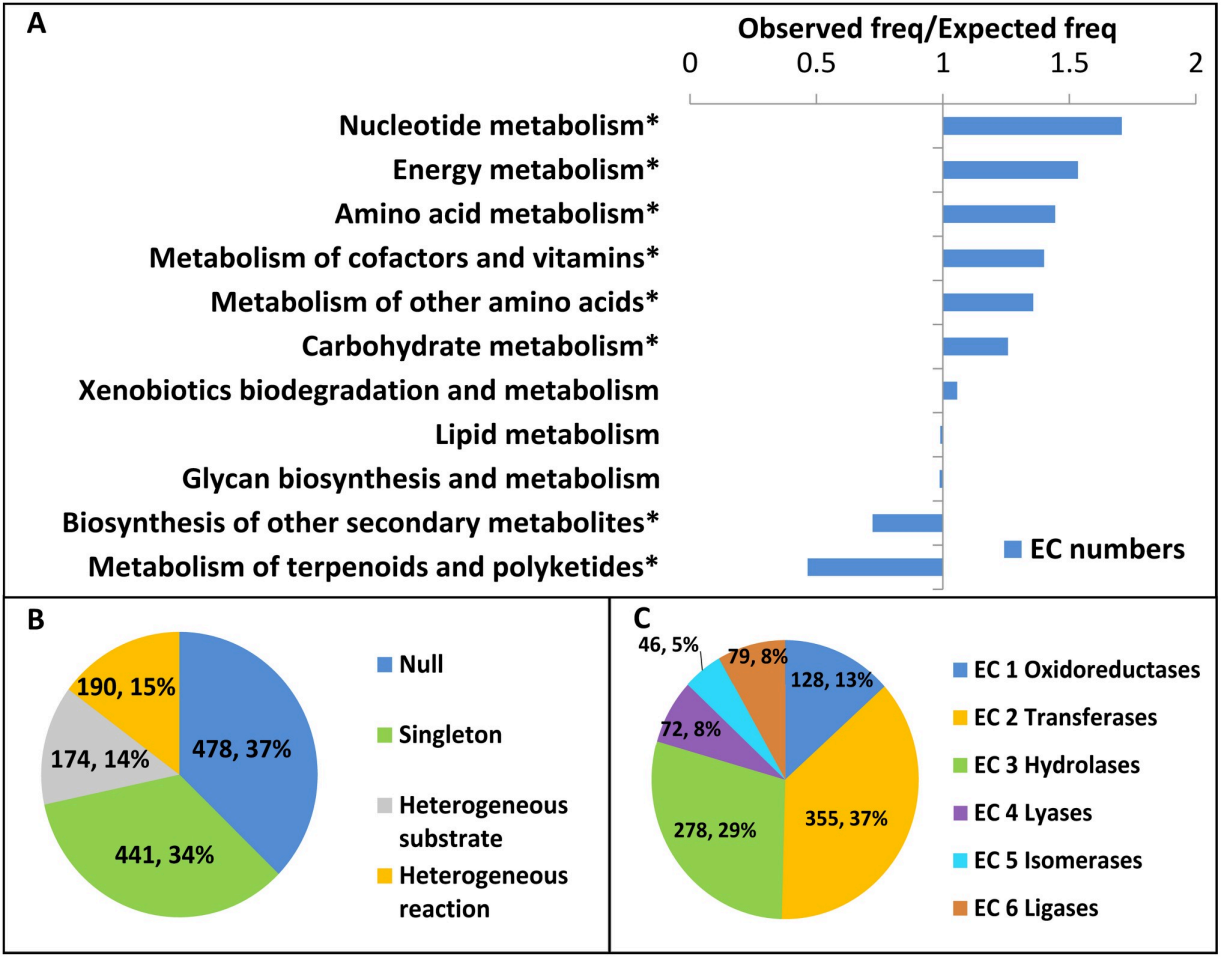
RLM enzymes reactions. **(A)** The ratio of observed and expected frequencies of metabolic reactions defines over (ratio>1) and under (ratio<1) represented pathway categories for RLM enzymes. Asterisks denote significant values according to Fisher’s exact test (P < 0.05). **(B)** Reaction categories of ECOD RLM families are depicted as a pie chart: families without any assigned EC reactions (Null), families with a single EC reaction type (Singleton), families with similar chemistries, but different substrates (Heterogeneous substrate), and families with different chemistries (Heterogeneous reaction) **(C)** Distribution of EC reaction classes among all RLM ECOD families that have an EC number assignment.

Inspection of RLM-involved pathways suggests that although all major metabolic pathways are represented in the RLM enzymes reactome, some categories are overrepresented. We calculated the observed frequency for RLM-involved metabolic reactions in each major category and compared it to the expected frequency for all reactions in that category to highlight over and underrepresented pathways (Fig 2A). Reactions catalyzed by these proteins are significantly overrepresented in nucleotide metabolism. RLM-involved reactions represent 49 out of 110 reactions in purine metabolism and 31 out of 65 reactions in pyrimidine metabolism. Similarly, RLM enzymes are significantly overrepresented in energy metabolism, including reactions in the ancient Wood-Ljungdahl pathway to form Acetyl-CoA [30] (5 out of 8 reactions in the pathway). Finally, RLM-involved metabolic reactions are significantly underrepresented in the biosynthesis of other secondary metabolites and the metabolism of terpenoids and polyketides. Polyketides are secondary metabolites of extremely diverse chemical structure. They are synthesized by enzymatic assembly lines that are typically encoded by large gene clusters. The underrepresentation of RLM domains in such pathways might reflect the fast-evolving nature of these enzymes and the difficulty in assigning the catalytic activity for each component in the enzymatic assembly lines.

Highlighting the conservative nature of EC assignments [31], we find that many ECOD families possess catalytic roles that have not been assigned EC numbers (i.e. 91 families have a UniProt or GO annotated active site, catalytic activity, or cofactor). Mapping of chemical reactions to RLM containing ECOD families (Fig 2B) showed that 37% (478 out of 1259) of the F-groups have no EC assignment (i.e. a null assignment), and many of these families are suggested to bind nucleotide by other databases (i.e. 82 families have UniProt annotated nucleotide binding or GO annotated molecular function ATP, GTP or nucleotide binding). Other non-catalytic roles for RLM structures include cellular transport, DNA binding, and structural constituents of the ribosome.

### RLM homologs can catalyze diverse reaction types

Rossmann folds are ancient, frequently diverged domains that participate in all known enzymatic reaction categories (Fig 2C). Unsurprisingly, the overall distribution of EC reaction classes among RLM ECOD families is in agreement with the statistics for all known proteins with 3D structures available in the PDB (https://www.rcsb.org/stats/enzyme). For both datasets the most prevalent enzymatic classes are oxidoreductases, transferases, and hydrolases. Thus, the functional diversity of RLM enzymes, which cover the six major classes of catalytic reactions (by the EC top-level numbering scheme), agrees with their structural and evolutionary diversity.

Among the RLM structures with assigned chemical reactions, 34% (441 out of 1259) are in homogeneous reaction families acting on a single substrate; 14% (174 out of 1259) are in homogeneous reaction families with heterogeneous substrates; and 15% (190 out of 1259) are in heterogeneous reaction families (Fig 2B). Heterogeneous reactions tend to arise within larger RLM ECOD F-groups. The top three ECOD family groups with largest number of unique EC numbers are shown in Table 1. Detailed description of the Table 1 can be found in S2 Appendix. It should be noted that 28% (53 out of 190) of F-groups that contain different EC numbers are bi- or multifunctional proteins (i.e. the same chain of PDB structure has multiple assigned EC numbers), suggesting that proteins in these families may readily adapt different functions.

**Fig 3:**
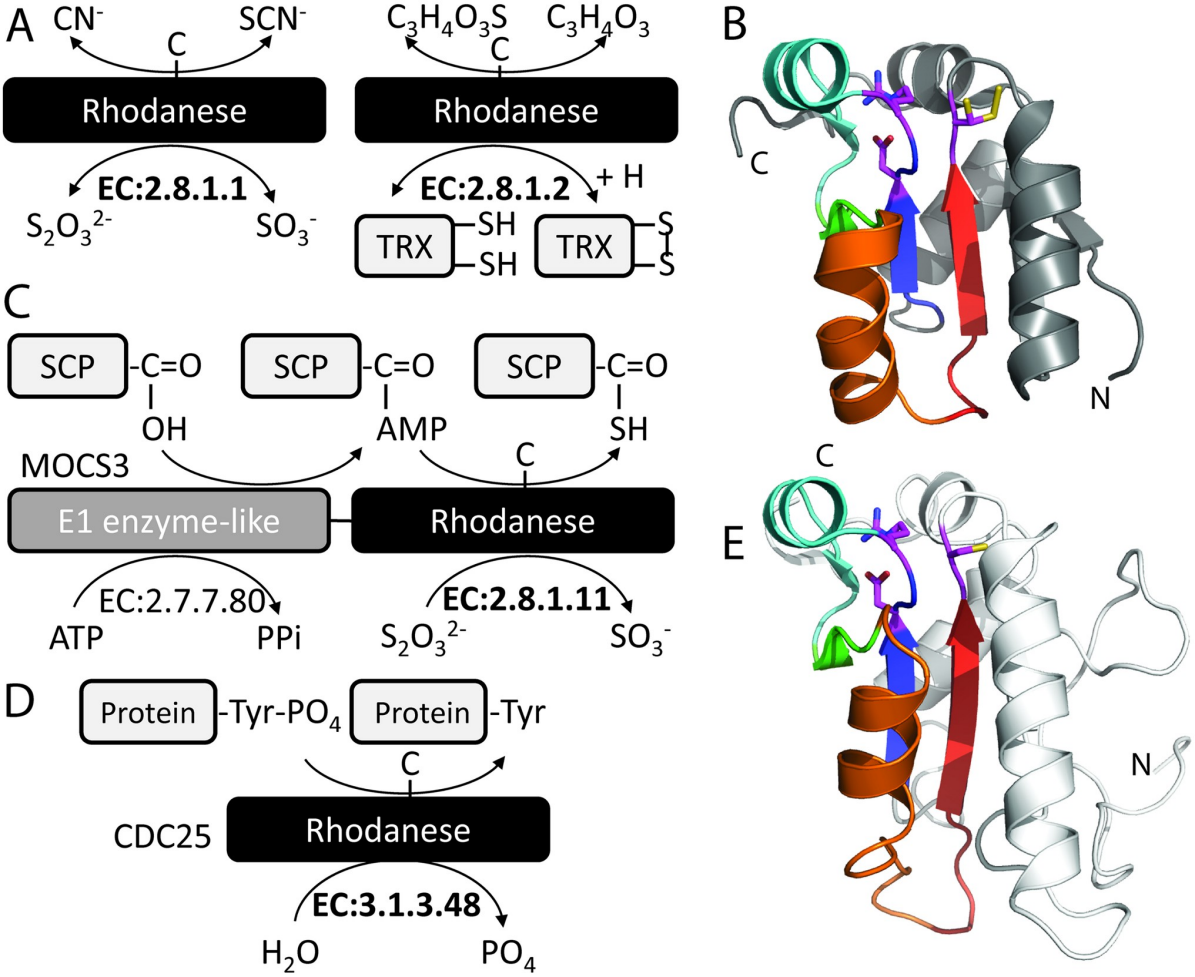
**Heterogeneous reactions catalyzed by the Rhodanese F-group** (EC numbers in bold) through Cys residue intermediates: **(A)** Rhodanese reactions transfer sulfur groups from thiosulfate to cyanide (EC: 2.8.1.1) or from mercaptopyruvate to oxidize thioredoxin (EC: 2.8.1.2). **(B)** GlpE rhodanese domain from PDB: 1GMX (gray cartoon) with RLM (rainbow) and active site with an additional conserved motif (magenta stick). **(C)** Sulfur group transfer to the C-terminal ampylated Gly residue of sulfur carrier protein (SCP) in a bi-functional MOCS3 (Molybdenum Cofactor Synthesis 3) enzyme (EC: 2.8.1.11 and EC: 2.7.7.80). **(D)** CDC25 phosphate hydrolysis from protein Phospho-Tyr residue (EC: 3.1.3.48). **(E)** CDC25 phosphatase rhodanese-like domain from PDB: 1QB0 with RLM domain (rainbow) retains similar active site (magenta stick). **(B, E)** Note that the β-strand (β2; green) can be considered as being barely existent (or vestigial) among the universe of RLMs being considered in this work.

The rhodanese family group (ECOD: 2007.2.5.2) exemplifies a heterogeneous reaction group catalyzing diverse reactions using similar active site chemistry (Fig 3). Rhodanase enzymes transfer the sulfur containing groups from thiosulfate to cyanide (EC: 2.8.1.1, Fig 3A) or from mercaptopyruvate to oxidize thioredoxin (MST; EC: 2.8.1.2) using an active site Cys residue [32]. The rhodanese active site Cys is in the loop following the last RLM β-strand (β3), while two additional conserved residues (motif DxR) are in the catalytic loop following β1 (Fig 3B). An additional rhodanese substrate is illustrated by an RLM domain (EC: 2.8.1.11) that transfers sulfur to the C-terminal glycine of an adenylated sulfur carrier protein (SCP). This domain is associated as a bifunctional protein with an E1 ubiquitin-activating domain (EC: 2.7.7.80) in molybdenum cofactor synthesis proteins (Fig 3C). Rhodanese-like family group domains also function as both serine/threonine (EC: 3.1.3.16) and tyrosine (EC: 3.1.3.48) protein phosphatases (CDC25 phosphatases, Fig 3D). The corresponding CDC25 phosphatase active site loop retains a CX5R motif (Fig 3E), with the invariant Cys forming a phosphointermediate in the reaction [33]. Thus, structures that belong to this relatively large rhodanase-like family catalyzes diverse reactions using a catalytic Cys to transfer either a sulfur group (EC: 2.8.1.-) or a phosphate group (EC: 3.1.3.-) from a small molecule or a protein substrate to a small molecule or a protein product.

### Converging to similar functions: Non-homologous RLM families can catalyze the same reactions

**Fig 4:**
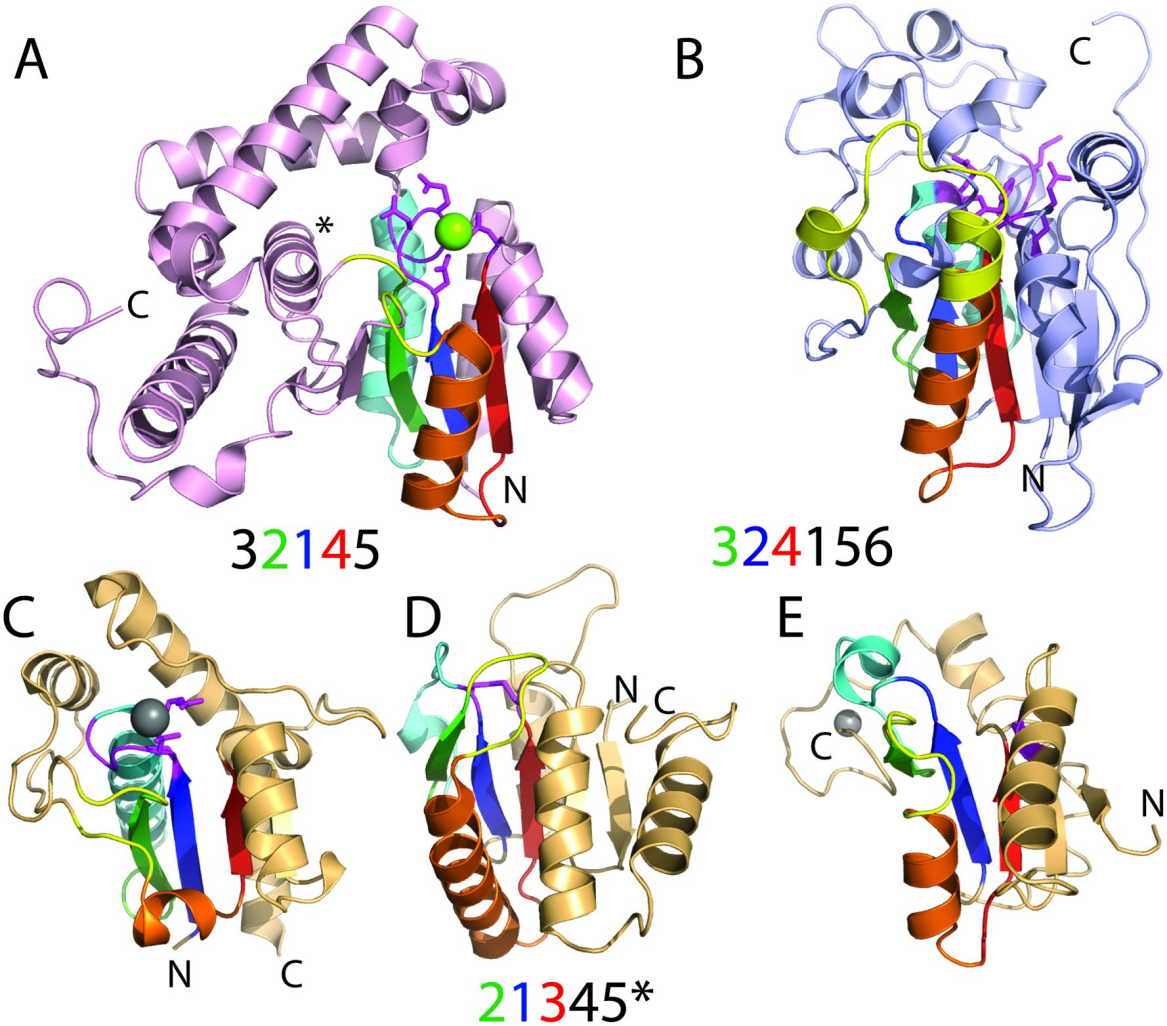
RLM protein phosphatases arose multiple times in evolution. Cartoon depictions are labeled at the termini. **(A)** HAD domain-like phosphatase (pink cartoon) adopts the parallel β-strand topology order indicated below and binds Mg^2+^ cofactor (green sphere) using the active site motif (magenta) from the RLM (rainbow). **(B)** PGM phosphatases (light blue cartoon) adopt the mixed β-strand topology order indicated below and catalyze metal-independent hydrolysis using a composite active site motif (magenta) that is both within and outside of the RLM (rainbow). Flavodoxin-like protein phosphatases (light orange cartoon, rainbow RLM, and magenta active site) fall into different ECOD T-groups with the typical flavodoxin-like β-strand order indicated below. The * denotes alternate T-group topologies for β-strand 5 (missing in panel C, permuted and antiparallel in panel D, and permuted in panel E). **(C)** Phosphotyrosine protein phosphatases I-like **(D)** (Phosphotyrosine protein) phosphatases II **(E)** rhodanese/cell cycle control phosphatase.

RLM enzymes can catalyze the same reactions using distinct topologies (Fig 4). The phosphoprotein phosphatase activity (EC 3.1.3.48) performed by rhodanese-like domains, which adopt a flavodoxin-like fold, is also catalyzed by RLM structures from non-homologous groups, including a HAD domain-like enzyme (ECOD: 2006.1.1.22) and a phosphoglycerate mutase-like (PGM) enzyme (ECOD: 2111.21.1.1). Each of these RLM containing phosphatases belong to different non-homologous X-groups in ECOD, have different core β-sheet topologies, and catalyze their protein phosphatase reactions using different mechanisms.

Phosphoryl hydrolase activity on protein substrates mediated by a HAD domain-like enzyme [34] uses a DxDE motif located in the RLM catalytic loop, together with a D following the last RLM β-strand β3 that coordinates Mg^2+^ (Fig 4A), to facilitate the formation of a phosphoenzyme intermediate that is then broken to release phosphate. Thus, the RLM contributes directly to the active site in the HAD domain, which adopts a five-stranded parallel β-sheet (order 32145) flanked by four helices, with a perpendicular α-helix following β-strand β2 (Fig 4A, marked by*). HAD enzymes with similar topologies use Mg^2+^ cofactors to catalyze phosphatase activity on small molecules (i.e. EC 3.1.3.77 and EC 3.1.3.18) or to catalyze other reactions (e.g. ATPase, dehalogenase, phosphosugar mutase, and phosphonatase). These reactions can be accommodated by small changes in HAD domain RLM active site, and the protein phosphatase activity is thought to have emerged in eukaryotes from HAD domains with alternate activity [35].

Alternately, the unrelated PGM family Sts-1 phosphatase (PDB: 2H0Q) uses a signature motif (RHGE) to catalyze metal-independent hydrolysis of phosphoproteins [36]. Sts-1 and other PGM family members adopt a 3-layer α/β/α sandwich with a six-stranded mixed β-sheet (order 324156, with β-strand 5 antiparallel to the rest). The RLM motif comprises β-strands 2–4, and the signature RHGE motif is located outside the RLM in the loop following β-strand β1. Additional catalytic residues are in the loop following the last RLM β-strand β3 and in the first RLM α-helix α1 (Fig 4B). In addition, PGM family enzymes with a similar fold hydrolyze phosphate from small molecule substrates like riboflavin-5-phosphate (EC 3.1.3.26) or inositol hexakisphosphate (EC 3.1.3.8).

Finally, the ECOD flavodoxin-like X-group includes several examples of evolutionarily-related phosphatases that exhibit different topologies (ECOD 2007.2.3.1, 2007.2.2.X, and 2007.2.5.2); a similar principle of “architectural similarity despite topological variability” is paralleled in the small β-barrel (SBB) domains [37], as articulated in the “urfold” concept recently advanced by Mura *et al*. [38]. Flavodoxin-like folds typically adopt a three-layer α/β/α sandwich with a five-stranded parallel β-sheet (order 21345). Different protein phosphatase T-groups substitute the canonical flavodoxin-like β-strand 5 with β-strands from alternate positions. The phosphotyrosine protein phosphatases I-like T-group (ECOD: 2007.2.2) exemplified by low-molecular-weight protein tyrosine phosphatase (LMWPTPase) retains the traditional flavodoxin-like topology but lacks the final β-strand (parallel sheet order 21345*, Fig 4C). LMWPTPase uses a PTP loop motif with Cys residues in the RLM catalytic loop to catalyze hydrolysis [39]. Alternatively, the phosphotyrosine protein phosphatases II T-group substitutes β-strand 5 with an anti-parallel β-strand insertion N-terminal to β-strand 4. Several different phosphatase families adopt this topology, including phosphatase PTEN (Fig 4D), which uses Cys residues from the RLM catalytic loop and the loop following the final RLM β-strand β3. The rhodanese/cell cycle control phosphatase T-group replaces flavodoxin-like β-strand 5 with an N-terminal extension. The plant dual specificity CDC25 phosphatase exhibits this topology, but the canonical rhodanese active site Cys residue is not in the loop following the RLM but rather in the following α-helix (magenta side-chain in Fig 4E).

### RLM enzymes bind a diverse set of ligands

**Fig 5:**
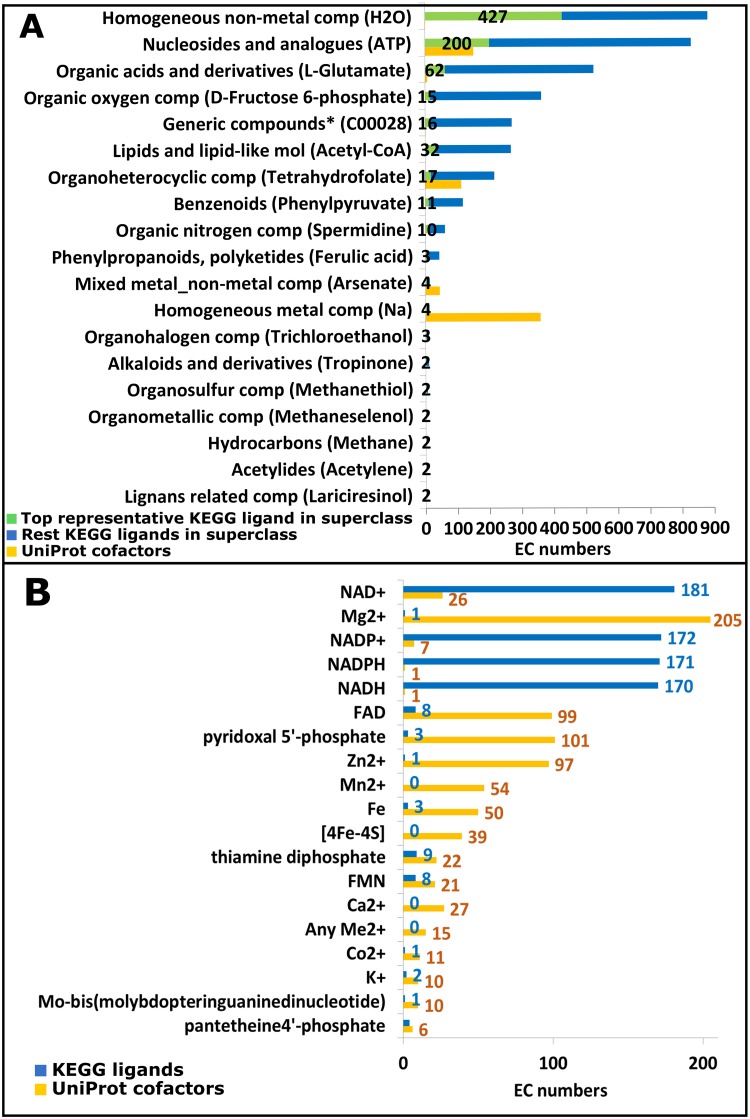
Ligands from RLM catalyzed reactions. **(A) ClassyFire superclasses** KEGG-defined compounds associated with RLM EC numbers reflect substrates and products of the reaction. Ligands populate 18 out of 31 ClassyFire superclasses and one additional unclassified group (marked by *). (**B)** UniProt KB and KEGG distinguishes RLM associated cofactors (41 organic and inorganic cofactor compounds. Top 19 compounds are shown).

RLM enzymes adopt a very large number of different functions, which implies binding of diverse ligands. Here we discus major superclasses of ligands and their binding modes for the top 10 most populated RLM H-groups. We collected all ligands (2468 total) associated with RLM EC numbers and defined as “Substrate” and “Product” from KEGG Compound [20] (13%–2468 out of 18505 metabolites and other small molecules, as of 2019/3/12) and as “Cofactor” from UniProt KB. Consistent with number of reactions catalyzed by the RLM [40], RLM ligands encompass a large and diverse set of chemical compounds (Fig 5A and 5B), and represent 18 out of 31 ClassyFire superclasses [11] (S5 Table: http://prodata.swmed.edu/rossmann_fold/lig_class/).

**Table 2:** Major binding modes of the 10 most populated RLM H-groups in ECOD.

ECOD X-group	ECOD H-group	ECOD F-group	PDB rep	EC number	Ligands cluster type	Binding site
Rossmann-like	2003.1	2003.1.1.35	1up6A	3.2.1.86	mostly nucleotides and their analogs	α1 binds phosphate and an Asp/Glu motif at the C-terminal end of β2, binds ribose from various cofactors including S-adenosylmethionine (SAM), NAD and FAD
					heterogeneous	adjacent to the nucleotides cluster, interacting with the loops and α-helices following β3
	2003.6	2003.6.1.2	2r3bA	4.2.1.136	mostly nucleotides and their analogs	binding site C-terminal to the RLM that is adjacent to the typical Rossmann-like site
					mostly organoheterocyclic compounds (PLP) and organic oxygen compounds (saccharides)	bind to the catalytic loop, α-helix α1 and the loop after β3 and represents the substrate (PLP or sugar) that gets phosphorylated by ADP/ATP in the adjacent nucleotide binding site
Flavodoxin-like	2007.1	2007.1.14.1	2xvyA	4.99.1.3	mostly nucleotides and their analogs	interacts with RLM β3 and the following loop. A second site of ATP and its derivatives, is formed by RLM catalytic and crossover loops and the α-helix following β3
					mostly organoheterocyclic compounds (heme and cobalamin)	interacts with the catalytic loop, α1 and loop following β3
	2007.2	2007.2.1.5	1sg0A	1.10.5.1	mostly nucleotides and their analogs	interacts with RLM catalytic loop, α1, β3 and non-RLM elements at the C-terminal part of the domain
Other Rossmann-like structures with the crossover	2111.6	2111.6.1.12	1s4pA	2.4.1.-	mostly nucleotides and their analogs	interacts with side chains from α-helix α1 and loops C-terminal to the RLM
	2111.77	2111.77.1.28	4a0gA	2.6.1.62 6.3.3.3;	mostly organoheterocyclic compounds (PLP)	PLP forms a Schiff-base with a lysine from a loop C-terminal to the RLM and binds at an RLM dimer interface. Catalytic loop and β3 bind the pyridoxal ring, a α-helix cap and loops C-terminal to the RLM bind the PLP phosphate
					mostly organic acids and derivatives	interacts with RLM α1 but primarily bind to the non-RLM C-terminal domain
P-loop domains-like	2004.1	2004.1.1.64	1g64A	2.5.1.17	mostly nucleotides and their analogs	mostly ATP and GTP, whose phosphates bind to this P-loop motif between β1 and α1
HUP domain-like	2005.1	2005.1.1.126	1g8gA	2.7.7.4	mostly nucleotides and their analogs	interacts with RLM β1, catalytic loop, α1 and β3 and the following loop
HAD domain-like	2006.1	2006.1.1.6	2c4nA	3.1.3.5	metal cations	binds between the catalytic loop and the loop after β3 and represent a common metal chelation site among HAD enzymes
					mostly organic oxygen compounds	interacts with loop after β2 and helical insertion after β1
					heterogeneous	interacts with β1, the catalytic loop, and the loops after β2 and β3
Phosphorylase/hydrolase-like	2011.1	2011.1.1.11	1rtqA	3.4.11.10	heterogeneous: organic acids and derivatives, organoheterocyclic compounds, metal cations, benzenoids	interacts with the catalytic loop, crossover loop, loop after β3, and some of the C-terminal structural elements

Comparison of ligand superclasses identified as substrates/products by KEGG with those cofactors assigned by UniProt highlights several key differences (Fig 5A, blue/green vs. yellow bars). Homogenous metal compounds (e.g. Mg^2+^) and mixed metal compounds (e.g. Fe-S) tend to function predominantly as cofactors in reactions with RLM enzymes but not substrate or products. These metal/mixed metal compounds aid in chemical catalysis, for example by acting as Lewis acids that activate a moiety (i.e. Zn^2+^ in Zn-dependent exopeptidases) or properly positioning substrates (i.e. Mg^2+^ bound to ATP in P-loops). They can also function in allosteric regulation or by stabilizing enzyme structure, both of which are discussed in examples below (see section “Diverse inorganic cofactor binding modes in RLM enzymes”).

Additional non-metal organic compounds, such as FAD, pyridoxal phosphate, NAD+, NADP+, NADH, NADPH and thiamine diphosphate, are ligands in numerous RLM enzymes (Fig 5B). While organic compounds mainly act as cofactors, some are substrates or products of RLM enzymes (e.g. EC 2.7.6.2, thiamine pyrophosphokinase or EC 2.7.7.2, FAD synthetase). Additionally, while NAD and NADP are the most frequently observed RLM ligand in KEGG and is generally described as an essential enzyme cofactor in literature [41], its distinction as either a substrate or cofactor remains ambiguous. While authorities on enzyme catalysis define NAD and NADP as a cofactor [42], its inclusion in UniProt as a cofactor remains limited (Fig 5B).

Pyridoxal 5'-phosphate (PLP), the active form of vitamin B6, belongs to the "Organoheterocyclic compounds" superclass and is the most popular organic cofactor in our data set according to UniProt – 101 enzymes utilize it as cofactor. These enzymes belong to five ECOD homology groups and 16 family groups. PLP mostly takes part as a coenzyme in all transamination reactions (EC: 2.6.1.X; 39 of 101 EC numbers in our data set) and in certain decarboxylation (EC: 4.1.1.X; 12 of 101), deamination (EC: 3.5.99.X; 1 of 101), and racemization (EC: 5.1.X.X; 3 of 101) reactions of amino acids [43]. Some RLM enzymes also catalyze metabolism of PLP, such as pyridoxal phosphatase (EC 3.1.3.74) or pyridoxal kinase (EC 2.7.1.35).

The most prominent ligand superclass (Fig 5A) contains homogeneous non-metal compounds (e.g. water, orthophosphate and carbon dioxide), nucleotides and their analogs (e.g. ATP, NAD and FAD), and organic acids and derivatives (e.g. L-glutamate and acetic acid). Not surprisingly, the top ligand superclass includes water (427 reactions) as this core metabolite contributes to over 2000 chemical reactions defined by KEGG. Together with carbon dioxide, these two metabolites represent the only homogeneous non-metal compounds that are widely considered to be present on early Earth and are thought to belong to an ancient metabolism that precedes the availability of phosphate [44].

The prevalence of nucleotides and their analogs among RLM enzyme reactions is expected. RLM enzyme chemistries are dominated by nucleotides and their derivatives (see section “Evolution of nucleotide/nucleoside ligand binding in RLM enzymes: binding modes reflect homology groups”), as 80 ECOD homology groups utilize these compounds (S8 Fig: http://prodata.swmed.edu/rossmann_fold/lig_online_fig2/), including two of the largest and oldest topology groups: P-loop NTPases (ECOD: 2004.1.1) and classic NAD(P)-binding Rossmann-like folds (ECOD:2003.1.1) [45].

**Fig 6:**
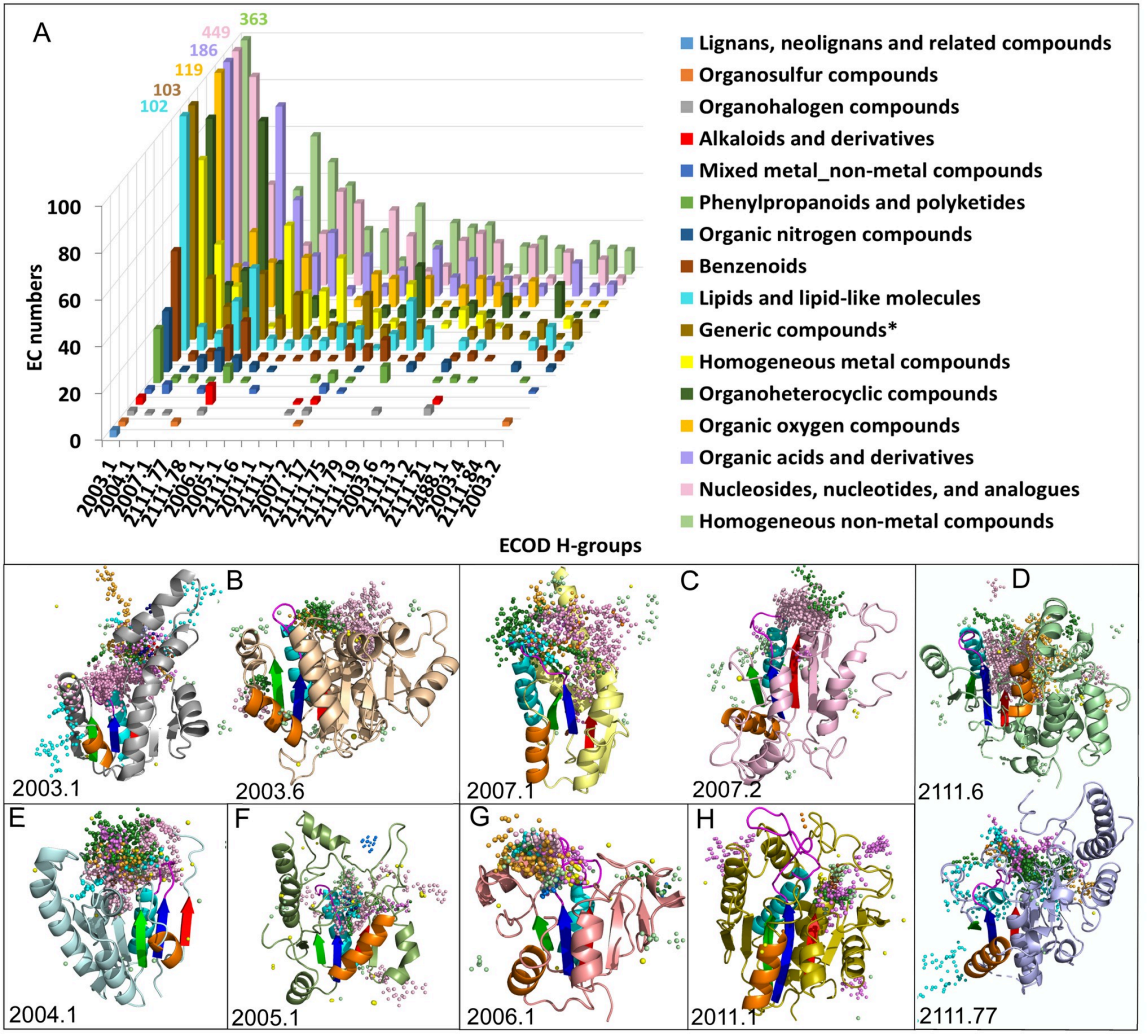
**(A) Ligands from RLM catalyzed reactions.** Combined KEGG compounds and UniProt cofactors (colored and classified according to ClassyFire superclass in legend) by assigned EC reaction count (Y-axis, cutoff at 100, with total number for EC>100 indicated) are distributed across top 23 ECOD homology groups (X-axis). **(B-H)** Superpositions of representative domains from largest H-groups. Secondary structure shown only for one representative. RLM is colored in rainbow. Ligands shown as spheres. Each sphere corresponds to one atom of particular ligand. Spheres are colored according ligands superclasses in legend of panel A.

Overall, our data show that RLM domains bind an unprecedented variety of ligands. For the top 10 most populated RLM H-groups that bind different ligand supercalsses (Fig 6A), we superimposed representative domains with different types of ligands to reveal binding sites that are inherent for this group (see Materials and methods). Fig 6B–6H illustrates these superpositions with ligand atoms colored according to ClassyFire superclass. Detailed descriptions of Fig 6 binding modes are given in S3 Appendix.

Table 2 summarizes major binding modes of the top 10 most populated RLM H-groups.

The top 10 most populated RLM H-group representative reveal that major binding sites inside RLM are in consistent with conserved positions shown in Fig 1C.

The doubly wound superfold, which encompasses the broad spectrum of RLM domains, was first identified as one of nine highly populated fold types [46]. This concept of a superfold was originally introduced to account for analogous folds that lack features suggestive of homology. Such analogous folds can retain similar binding sites, or supersites, presumably due to structure properties such as the α-helix dipole utilized by many Rossmann-folds [47] and H-bonds contributed by the N-terminus of an α-helix in some TIM barrels [47,48]. Indeed, ligand-binding supersites have been identified in seven of the nine superfolds [49]. While structure and chemical evidence suggest that a majority of the members of the TIM barrel superfold arose from a common ancestor [48, 50], evidence uniting RLM domains is less clear.

Like RLM domains TIM barrels are involved predominantly in metabolism, binding many diverse cofactors to catalyze five of the six major chemistry types [50]. However, the constraints placed on the circular TIM barrel topology tend to limit its structure diversity when compared to RLM domain folds, which can swap β-strand order and add or remove β-strands from either end of the β-sheet. The split α/β sandwich superfold is also characterized by a β-sheet that can extend on either side. Similar unclear evolutionary relationships exist for these folds, which can be exemplified by the large number of superfamilies existing in the alpha beta plaits X-group in ECOD (124 H-groups).

The diversity of RLM folds is also reminiscent of a set of simple structurally related domains known as the small β-barrels (SBB) [37]. The SBBs are thought to be robust in terms of fold stability, and thus can accommodate diverse sequence in loops and as extensions to the termini. SBBs utilize this plasticity, the ability to adopt higher order oligomers, and to serve as modules in multidomain proteins to generate functional diversity, especially in binding nucleic acids. Like the SBBs RLM folds can adopt higher order structures (i.e. hexameric rings for P-loop ATPases) and participate in multidomain protein function (i.e. 7 domains in isoleucyl-tRNA synthetase). However, a majority of RLM domains act alone or as dimers. Potentially, the larger size of RLM domains might limit their association into higher order complexes. All examples discussed in this work possess an RLM that is incorporated with the domain’s structural core and usually takes part in ligand binding. For large domains that extend the β-sheet, insertions to the RLM can occur between β2 and α2, or as extensions following β3. These insertions or extensions usually cooccur with the ligand-binding position (Fig 6B, 2003.6 and Fig 6C, 2007.2 have extensions to the C-terminus that surround the majority of ligand-binding site). Whereas, for small domains the RLM may constitute the majority of the core. Some representatives from the P-loop H-group contain two RLMs in a single domain and they sometimes retain common SSEs, namely β2 element of one RLM is β3 element for another one.

### Diverse inorganic cofactor binding modes in RLM enzymes

Many enzymes bind metal cations that either stabilize protein structure or facilitate chemical reactions. The divalent cations Mg^2+^, Ca^2+^ and Zn^2+^ are the most common metal elements utilized by enzymes in non-redox catalytic reactions [51]. Metalloenzymes frequently employ Fe, Cu and Mo as redox-active metals, where Fe is especially common because it is suitable for different types of reactions (such as electron transfer by cytochromes) as well as its availability over the course of evolution [52]. Metalloenzymes can mediate a wide range of chemical reactions, as their structural diversity permits access to multiple electronic configurations of metal centers [53]. Living systems have also evolved to synthesize enzymes that employ different metals for the same task.

Among the 402 RLM enzymes (by EC numbers) in our dataset with metal cofactors assigned by UniProt KB, 263 are present in the PDB structure. The most commonly observed metal cations (Mg^2+^, Zn^2+^, Mn^2+^, Fe^2+/3+^ and Ca^2+^) recount the most common metal cofactors utilized in biological systems. For the most common metal cofactor Mg^2+^, RLM domains appear to have evolved multiple different structural contexts (41 ECOD H-groups) and catalytic activities (205 EC numbers). These activities span all major reaction classes, with a majority corresponding to transferases (88 reactions) and hydrolases (52 reactions).

**Fig 7:**
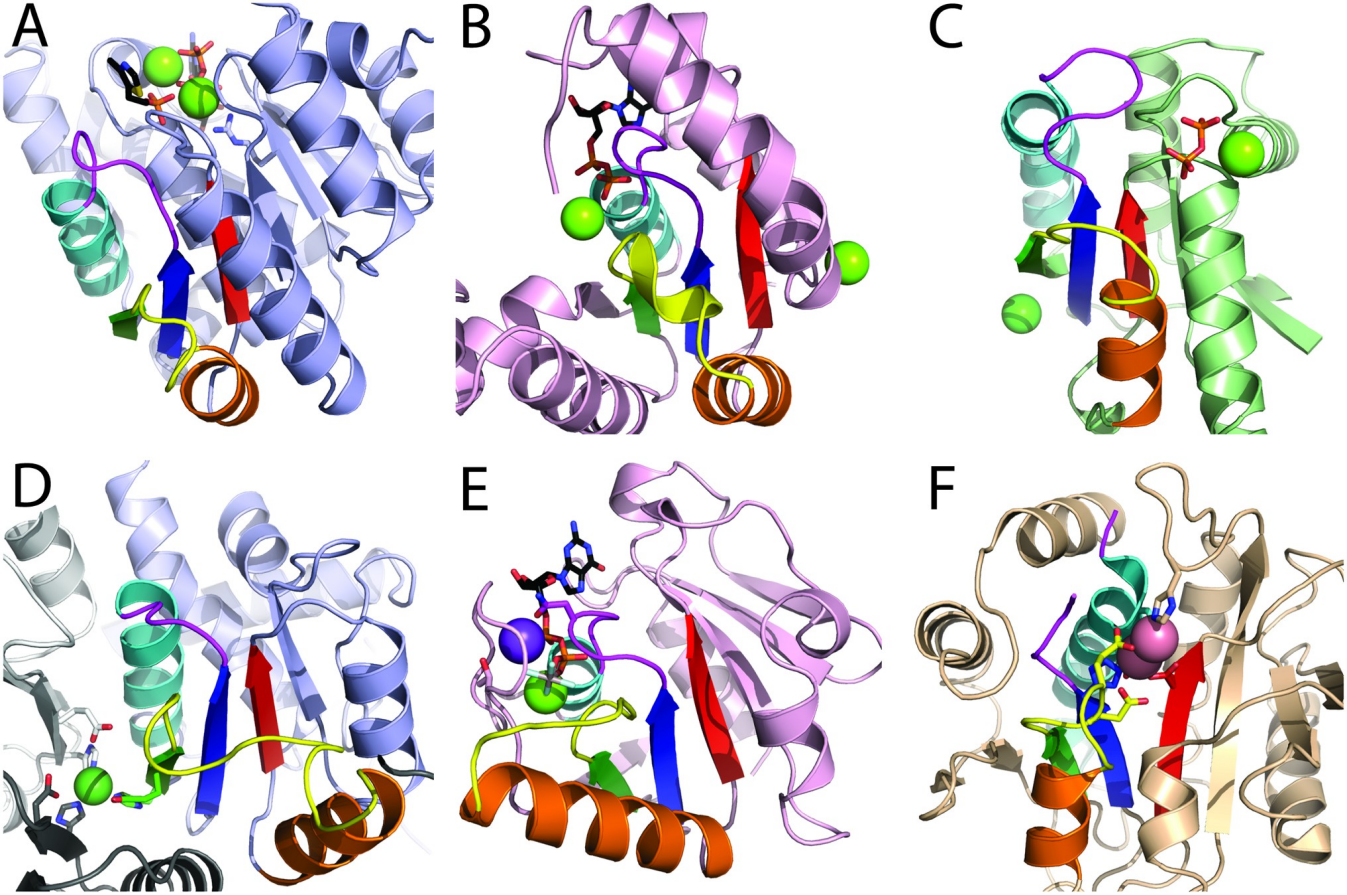
Diverse binding modes of inorganic metal cofactors. RLM SSEs are colored in rainbow with yellow crossover loop and magenta catalytic loop. **(A)** Mg^2+^ (green sphere) bound to 4-methyl-5-beta-hydroxyethylthiazole kinase (PDB: 1ESQ) Rossmann-like X-group domain (slate cartoon) coordinates ATP substrate (black stick, colored by element). **(B)** Mg^2+^ (green sphere) bound to shikimate kinase (PDB: 2SHK) P-loop domains-like X-group (pink cartoon) coordinates ADP (black stick, colored by element). **(C)** Mg^2+^ (green sphere) bound to sphingosine kinase (PDB: 3VZD) flavodoxin-like X-group domain (green cartoon) coordinates pyrophosphate (orange stick). (**D**) Ni^2+^ bound to trimer of ornithine transcarbamylase (PDB: 2W37) Rossmann-like X-group domain (slate cartoon) mediates trimerization (white and gray cartoon chains) through coordinating residues (stick). (**E**) MnmE G-domain (PDB: 2GJ8) from P-loop domains-like X-group (pink cartoon) binds K^+^ (violet sphere) near the transition state analog Mg2+ (green sphere)-GDP-AlF (black stick, colored by element). (**F**) Co^2+^ (pink spheres) bound to active site of cobalt-activated peptidase TET1 (PDB: 2CF4) phosphorylase/hydrolase-like X-group domain (wheat cartoon).

Five ECOD X-groups act as transferases utilizing Mg^2+^, including Rossmann-like (ECOD: 2003), P-loop domains-like (ECOD: 2004), HUP domain-like (ECOD: 2005), flavodoxin-like (ECOD: 2007), and “Other Rossmann-like structures with the crossover” (ECOD: 2111). Another four X-groups utilizing Mg^2+^ are kinases, transferring phospherous-containing groups to an alcohol group as an acceptor (EC 2.7.1.-). RLM enzymes coordinate Mg^2+^ in several different modes. For example, 4-Methyl-5-beta-hydroxyethylthiazole kinase (ThiK, PDB: 1ESQ) functions as a homotrimer and uses two Mg^2+^ cofactors to bind an ATP substrate at the subunit interface. Elements of the RLM form the binding site for the thiazole (substrate) in ThiK, while an adjacent loop and β-strand to the C-terminus of the RLM binds ATP and Mg^2+^ (Fig 7A). In contrast, shikimate kinase (PDB: 2SHK, A:1–170) coordinates the Mg^2+^ cofactor and ATP using the canonical P-loop motif located at the N-terminus of the first RLM α1 (Fig 7B). The flavodoxin-like domain (ECOD: 2007) of sphingosine kinase (PDB: 3VZD, A:6–145) illustrates a third coordination state for Mg^2+^ in RLM domains. This domain binds a metal ion, forming the active site between the flavodoxin-like domain and a NAD kinase beta sandwich domain in the C-terminus, using the α-helix directly C-terminal to the last RLM β-strand (β3). The phosphates of the ATP substrate bind to the N-terminal cap of the same α-helix. Interestingly, if we consider the flavodoxin-like (ECOD: 2007) topology as a circular permutation of the P-loop (ECOD: 2004) shikamate kinase, then the flavodoxin phosphate-binding α-helix becomes analogous to the phosphate-binding α-helix in the P-loop RLM. Finally, another flavodoxin-like domain (ECOD: 2007) from 6-phospho-1-fructokinase (PDB: 3F5M) binds Mg^2+^ ATP at the interface created by an inserted domain belonging to the “other Rossmann-like structures with the crossover” X-group (ECOD: 2111) (Fig 7C). While the Mg^2+^ ATP binds mainly to the flavodoxin-like domain, the long RLM crossover loop in the inserted phosphofructokinase domain coordinates Mg^2+^ and completes the ATP binding site.

The remaining four cations (K^+^, Co^2+^, Ni^2+^, Cu^2+^) could be observed as cofactors for only a few enzyme families. For example, the Rossmann-like X-group domain of ornithine transcarbamylase (cOTC) binds Ni^2+^ with the β-strand β2. This Ni^2+^ stabilizes a unique trimeric conformation of the enzyme (Fig 7D) [54]. In fact, Grueninger *et al*. [55] have shown that the center of cyclic and dihedral oligomers with 3-fold or higher symmetry provides competent environments for the binding of metals. Alternatively, the G-domain of MnmE adopts a P-loop like fold (Fig 7E) and binds potassium near the GDP-AlF transition state analog. Potassium serves to reorient the catalytic machinery and enhance catalysis [56]. Finally, Fig 7F shows the RLM domain of the cobalt-activated peptidase TET1 (PhTET1) [57]. Cobalt cations, which replace the typical Zn^2+^ ions of related Zn-dependent exopeptidases, are bound by residues from RLM β1, α1 and crossover loop.

### Evolution of nucleotide/nucleoside ligand binding in RLM enzymes: binding modes reflect homology groups

**Fig 8:**
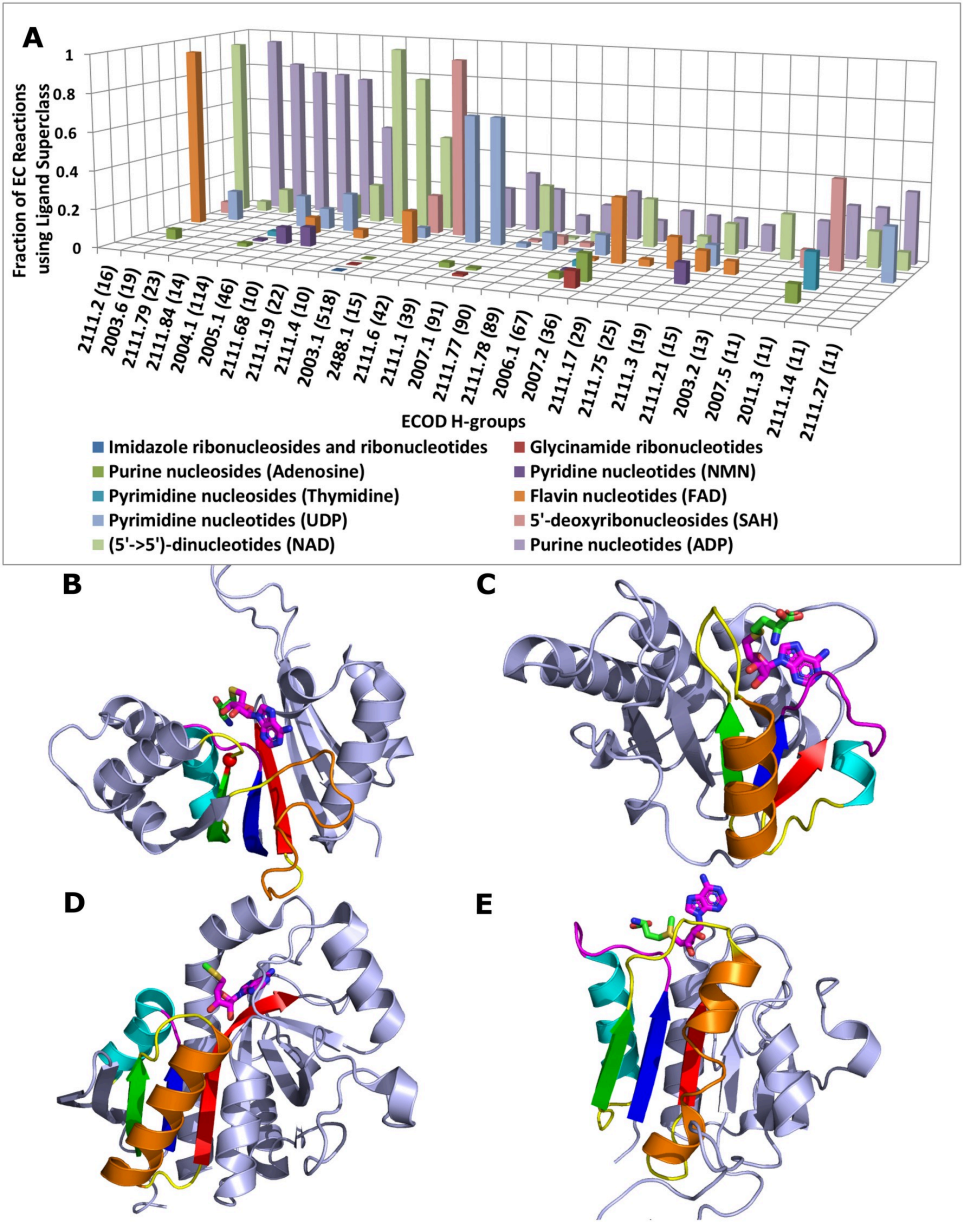
**(A) Distribution of ligands from “Nucleotide/nucleoside” superclass for H-groups with more than 10 EC reactions.** KEGG compounds (colored and classified according to classes of “Nucleotide/nucleoside” superclass in legend) by assigned fraction of EC reactions in particular homology groups (Y-axis) are distributed across ECOD H-groups with more than 10 unique EC numbers (X-axis). Values in parenthesis show the number of unique EC numbers per H-group. **(B-E) 5’-deoxyribonucleosides reveal different binding modes in different H-groups.** RLM (SSEs colored rainbow, with yellow crossover loop) bind adenine nucleotide (magenta stick) of SAH/MTA. 5’-deoxyribonucleosides-binding Rossmann-fold domain (slate cartoon) from **(B)** Mouse nicotinamide N-methyltransferase (PDB: 2I62, EC: 2.1.1.1) binds SAH, the Asp/Glu motif is shown by the red sphere **(C)** methyltransferase (PDB: 2CX8, EC: 2.1.1.193) binds SAH, the “knot” is formed by the blue β-strand, magenta loop, cyan α-helix and red β-strand with the following loop, which is located under the magenta loop, **(D)** MTA/AdoHcy nucleosidase (PDB: 1Z5O, EC: 3.2.2.9) binds 5'-methylthioadenosine (MTA), **(E)** 5'-fluoro-5'-deoxyadenosine synthase (PDB: 1RQP, EC: 2.5.1.63) binds SAM.

Homology frequently implies similarity of proteins’ binding sites. Here we show that RLM enzymes from different H-groups exhibit different binding modes for the same ligand. ClassyFire divides the “Nucleosides, nucleotides and analogues” superclass into 11 classes. The distribution of ligands from these classes among ECOD homologous groups (Fig 8A, 10 classes shown in 27 ECOD H-groups with the most EC numbers) highlights the evolutionary plasticity of the RLM enzymes towards catalysis (some H-groups catalyze over 500 reactions) as well as their tendency to utilize purine nucleotide ligands. Purine nucleotides, with ADP as the most frequent representative, are ligands for the most populated H-groups (65 H-groups out of 168, 41%). Additionally, the adenine component of ADP is present in other well-represented nucleotides, including ATP, NAD, FAD, and SAH. This wide distribution could reflect any or all of the following characteristics: the simplicity of adenine binding modes in protein architectures [58], the preponderance of adenine-containing ligands in extant biochemical reactions (including those that provide energy), and/or the exploitation of adenylate-binding motifs early in evolution as components of ancient proteins or ribozymes from an RNA world [59].

The Rossmann-related H-group (ECOD: 2003.1) has the largest number of distinct EC numbers among all H-groups. Domains from this group bind seven of ten nucleotide classes (Fig 8A). S-adenosyl methionine (SAM) and its derivative S-adenosyl-L-homocysteine (SAH) are among the most frequent ligands of proteins in this H-group, and they are important cellular methylating agents. An important structural feature of these proteins is the Asp/Glu motif at the tip of the β2, which takes part in ligand binding and plays an important role in binding the ribose component of SAH (Fig 8B) [60]. Most SAH-binding enzymes from this H-group function as different types of methyltransferases. These ligands provide methyl groups to a variety of acceptors including DNA, RNA, proteins, lipids, and various small molecules [61]. Binding modes shown in Fig 8 are discussed in details in S4 Appendix and are summarized in Table 3.

**Table 3:** Binding modes of 5’-deoxyribonucleosides class for major H-groups.

ECOD X-group	ECOD H-group	ECOD F-group	PDB	Protein name	EC	Ligand	Binding
Rossmann-like	2003.1	2003.1.5.54	2i62_A	Nicotinamide N-methyltransferase	2.1.1.1	SAH	SAH adenine ring interacts with crossover residues. Ribose ring binds to a G-rich motif in the RLM catalytic loop and last residue of second RLM β-strand β2. Homocysteine interacts with α1
alpha/beta knot	2488.1	2488.1.1.11	2cx8_A	Methyltransferases	2.1.1.193	SAH	Adenine ring binds in parallel plane to the β-sheet and interacting with catalytic loop and C-terminal of the β3. The ribose ring interacts with β1 and the crossover loop
Phosphorylase/hydrolase-like	2011.2	2011.2.1.6	1z5oA	MTA/AdoHcy nucleosidase	3.2.2.9	MTA	The adenine ring plane adopts a 45° angle relative to the plane of the middle β-sheet and interacts with the extended portion of the β3. The ribose ring interacts with catalytic and crossover loops, and residues C-terminal to the RLM.
Other Rossmann-like structures with the crossover	2111.37	2111.37.1.1	1rqpA	5'-fluoro-5'-deoxyadenosine synthase	2.5.1.63	SAM	The adenine ring plane is parallel to the β-sheet of the domain and interacts with crossover loop. The homocysteine carboxyl component interacts with the crossover loop and α-helix α1.

Our data demonstrate that non-homologous RLM enzymes adopt different binding modes for the same ligands from the nucleotides superclass (SAM, SAH, MTA). A specific binding mode is a distinctive feature of each ECOD H-group and presents important evidence for homology between the member domains. Different H-groups that bind the same ligand using different mode may perform the same function (e.g. RLM methyltransferases are the result of convergent functional evolution) or different functions.

### Divergent evolution of nucleotides/nucleosides binding sites

**Fig 9:**
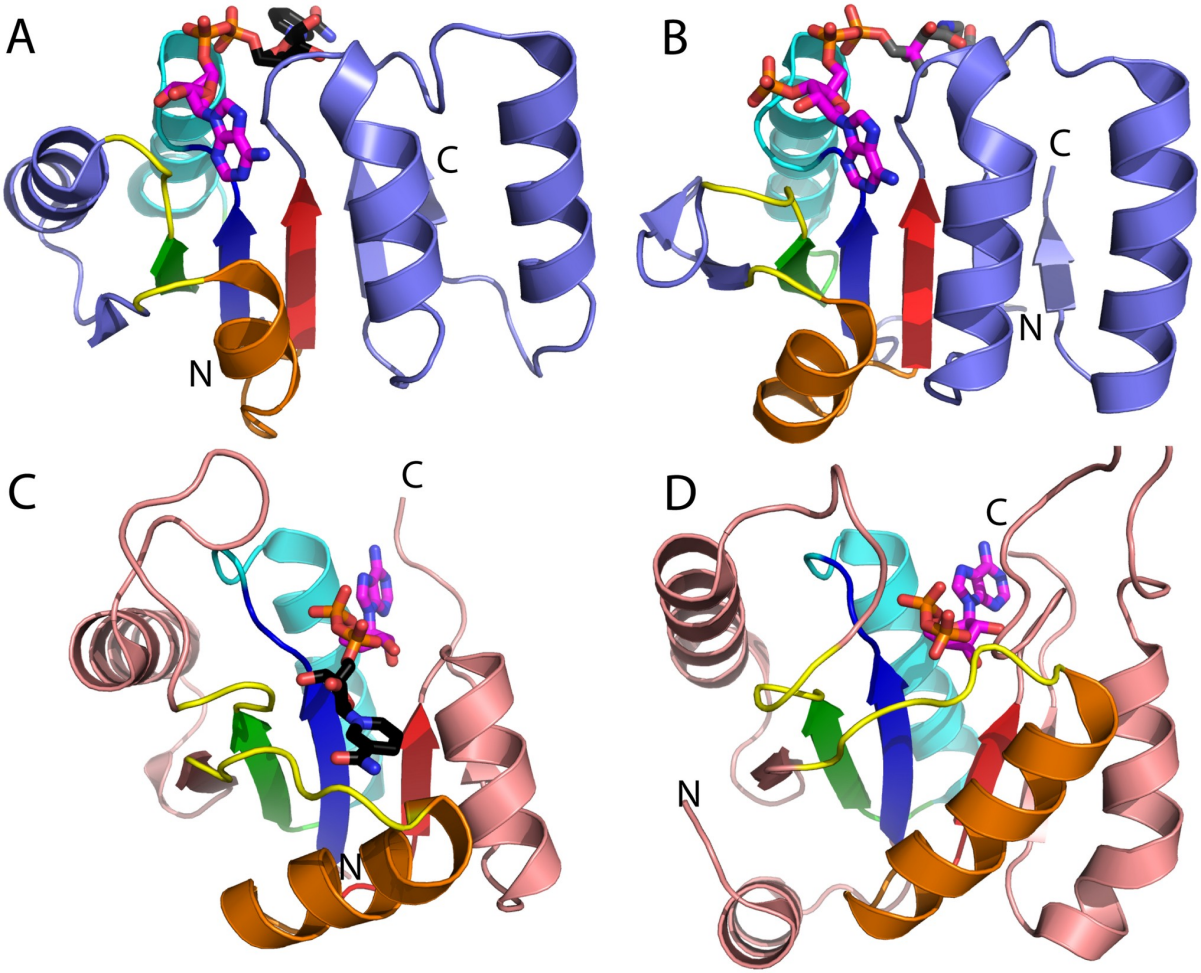
Binding site divergence in RLM homologs. RLM (SSEs colored rainbow, with yellow crossover loop) from homologs bind adenine nucleotide (magenta stick) and diphosphate (orange stick) components of NAD (black nicotinamide ring) from different substrates (stick, colored elements) using similar binding modes. NAD(P)-binding Rossmann-fold domain (slate cartoon) from **(A)** myo-inositol dehydrogenase (PDB: 4MIN) binds NAD and from **(B)** succinyl-CoA Synthetase (PDB: 2NU8) binds CoA (gray cysteamine). HUP domains (salmon cartoon) from **(C)** nicotinamide mononucleotide adenylyltransferase (NMNATase) (PDB: 1EJ2) binds NAD and from **(D)** bi-functional ATP Sulfurylase-APS Kinase (PDB: 2GKS) binds ADP.

Several cases of homologous RLM enzymes reveal divergent evolution of binding sites to accommodate different ligands. Classic nucleotide-binding Rossmann-fold domains (ECOD: 2003.1.1), as exemplified by myo-inositol dehydrogenase of the GFO/IDH/MOCA family, bind the adenine nucleotide ring of NAD in a pocket formed by the conserved G-rich motif in the RLM catalytic loop and loops from the crossover (Fig 9A). The RLM α-helix α1 dipole may mediate interaction with the NAD diphosphate, with the nicotinamide ring extending towards the C-terminal portion of the domain. In a closely related CoA-binding domain from succinyl-CoA synthetase, the binding mode for the adenine ring and diphosphate from CoA (Fig 9B) are identical to that of NAD, suggesting the C-terminal pocket diverged to accommodate the CoA cysteamine tail. The divergence of these RLM domains (Fig 9A and 9B) from a common ancestor is supported by sequence similarity identified between the cores of each domain, including the RLM (HHPRED probability score 99%) [62]. In fact, several families with classic nucleotide-binding Rossmann-fold domains bind CoA either as a substrate, a product, or an inhibitor. For example, NADPH-dependent malonyl-CoA reductase (MCR, PDB: 4DPM) carries out a reaction cycle in which the NADP(+) cofactor and the substrate/product CoA successively occupy the same bispecific binding site [63]. Additionally, peroxisomal 2,4-dienoyl CoA reductase (PDB: 4FC6) binds NADP cofactor in the typical site and binds hexadienoyl CoA substrate alongside the cofactor using insertions to the Rossmann domain core C-terminal to the RLM. Finally, archaeal ketopantoate reductase (KPR, PDB: 5AYV), binds CoA in the NAD(P)H binding site as a competitive inhibitor to regulate CoA biosynthesis.

Similar divergence of ligand binding sites has occurred in RLM enzymes from the HUP homology group with characteristic HIGH motifs [1]. NAD binding in nicotinamide mononucleotide adenylyltransferase (NMNATase) domains from this H-group is mediated by the RLM, although the dinucleotide is flipped with respect to classic nucleotide-binding Rossmann-fold domains (Fig 9C). The RLM crossover loop binds the nicotinamide ring, a conserved Arg from the catalytic loop binds the diphosphate, and the first RLM α-helix α1 (containing the HIGH motif) forms the pocket for the adenine ring. Another member of the HUP H-group, ATP sulfurylase (Fig 9D) binds ADP with the same components as in the NMNATase binding site, except that the RLM crossover loop and following α-helix α2 close the nicotinamide pocket. As adenosine phosphates are a canonical ligand of typical HUP domains [1], this HIGH-motif-containing RLM domain likely evolved from an ancestral ATP binding site to accommodate NAD dinucleotide. Thus, our results exemplify divergent evolution of homologous RLM domains, which bind different ligands using similar binding modes.

### Allosteric nucleotides/nucleosides binding sites in RLM domains

**Fig 10:**
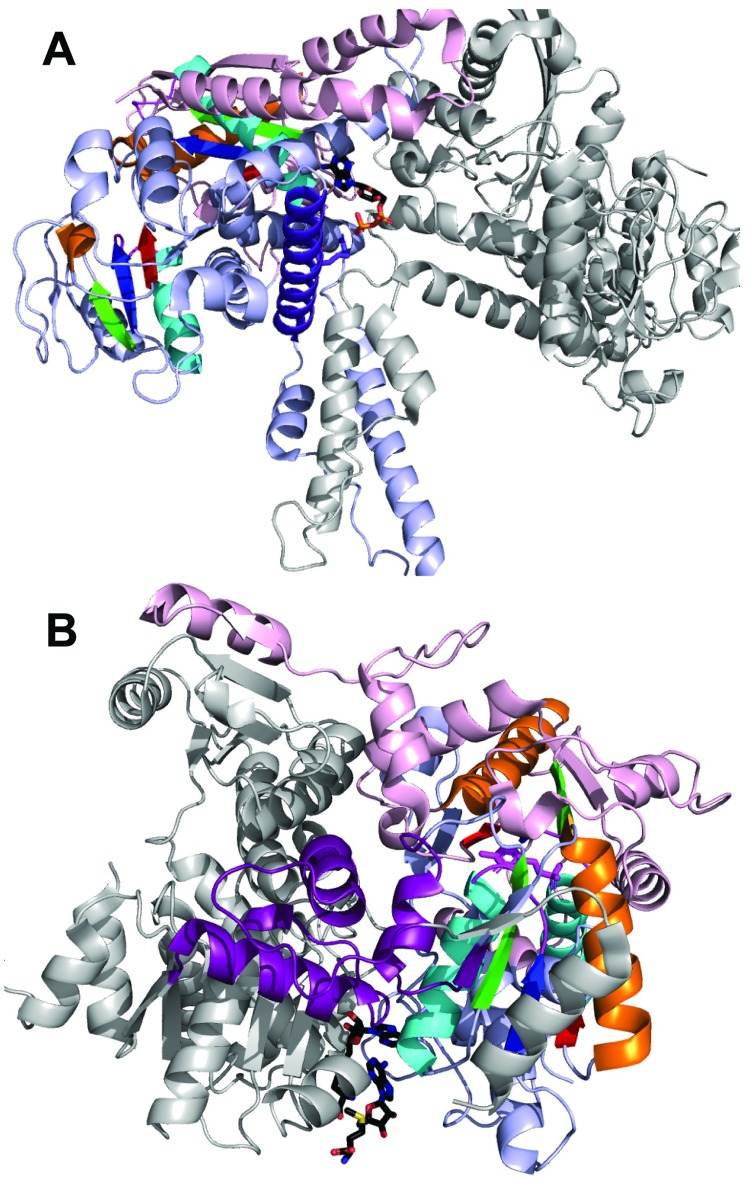
Nucleotides/Nucleosides allosteric binding. Examples of RLMs that do not mediate binding of ligands are shown in rainbow, with typical binding site loops in magenta. **(A)** ADP (black stick, colored by element) binds to bovine glutamate dehydrogenase (PDB: 1NQT) in the inter-chain interface (second chain in white). N-terminal flavodoxin-like domain is in light pink and C-terminal Rossmann-like domain is in light blue. Pivot helix is in purple. R459 shown by purple sticks. **(B)** Two SAMs (black stick, colored by element) bind to threonine synthase (PDB: 2C2B) interdomain interface distant from the typical Rossmann-like domain (N-terminal domain, light blue) active site marked by the PLP cofactor (magenta stick). C-terminal RLM domain is in light pink. The rigid block is in purple.

Nucleotides and their analogs can bind to various locations in RLM structures, including allosteric sites remote from the active site. RLM allosteric binding sites are prospective targets for drugs. The main advantage of allosteric drugs is “non-competitive action via modulation of protein structural dynamics” [64]. Bovine glutamate dehydrogenase (GDH) is one example of such allostery. GDH catalyzes the reversible oxidative deamination of L-glutamate to 2-oxoglutarate (EC:1.4.1.3). A GDH monomer consists of two RLM domains: an N-terminal “glutamate-binding” flavodoxin-like domain (ECOD: 2007.1.6.1, Fig 10A light pink) and C-terminal NAD binding domain (ECOD: 2003.1.1.24, Fig 10A light blue). A complex pattern of allosteric modulation of GDH has evolved [65], with GTP and NADH serving as allosteric inhibitors and ADP and NAD as allosteric activators. Fig 10A shows GDH with ADP bound in the dimer interface (PDB: 1NQT), which is removed from the active site formed by the RLM in the C-terminal NAD binding domain (Fig 10A, magenta active site in pink domain). ADP binding induces conformational change and NAD binding domain rotates down to initiate catalysis. This rotation occurs about a “pivot helix” at the C-terminal portion of the NAD binding domain (Fig 10A purple). C-terminal helices of this domain constitute an “antenna”, forming the catalytic mouth, that opens and closes during conformational change [66].

Threonine synthase (TS) provides a second example of allosteric binding by a ligand S-adenosylmethionine (SAM). TS is a fold-type II pyridoxal phosphate (PLP)-dependent enzyme that catalyzes the ultimate step of threonine synthesis (EC: 4.2.3.1) in plants and microorganisms. Unlike the microbial enzyme, plant TS is activated by SAM [67]. Two similar chains of the TS protein (PDB: 2C2B) form a dimer and bind four SAM ligands in tandem at the dimer interface (Fig 10B) remote from the PLP-containing active site of the RLM domain. Each dimer consists of two RLM domains from the same homology group (ECOD: 2003.4). Binding of SAM induces a sliding of both monomers along the dimer interface, which triggers a rotation of the N-terminal RLM domain (Fig 10B light blue) toward and of the C-terminal domain (Fig 10B light pink) away from the interface [67]. This shift occurs around a rigid block colored in purple in Fig 10B.

### Iron-sulfur cluster reactions

Iron-sulfur (Fe-S) clusters are ancient cofactors usually bound to cysteines of associated proteins [68]. These Fe-S clusters are often classified based on their cluster composition, as suggested by the Nomenclature Committee of the International Union of Biochemistry (IUB) [69]. In our data set, we observed seven types of iron-sulfur clusters bound to RLM domains: [2Fe-2S], [3Fe-4S], [4Fe-4S], [8Fe-7S], [Ni-Fe-S] and [Fe-O-S] hybrid clusters with different modifications, and [7Fe-Mo-9S]. In total, 44 of the RLM domain associated EC numbers from our dataset bind iron-sulfur clusters according to UniProt KB (see Materials and methods). 64% (28 out of 44) of the enzymes are oxidoreductases, 11% (5 out of 44) are transferases, 5% (2 out of 44) are hydrolases and 20% (9 out of 44) are lyases. 89% (39 out of 44) of all iron-sulfur proteins in our data set bind [4Fe-4S] clusters.

**Fig 11:**
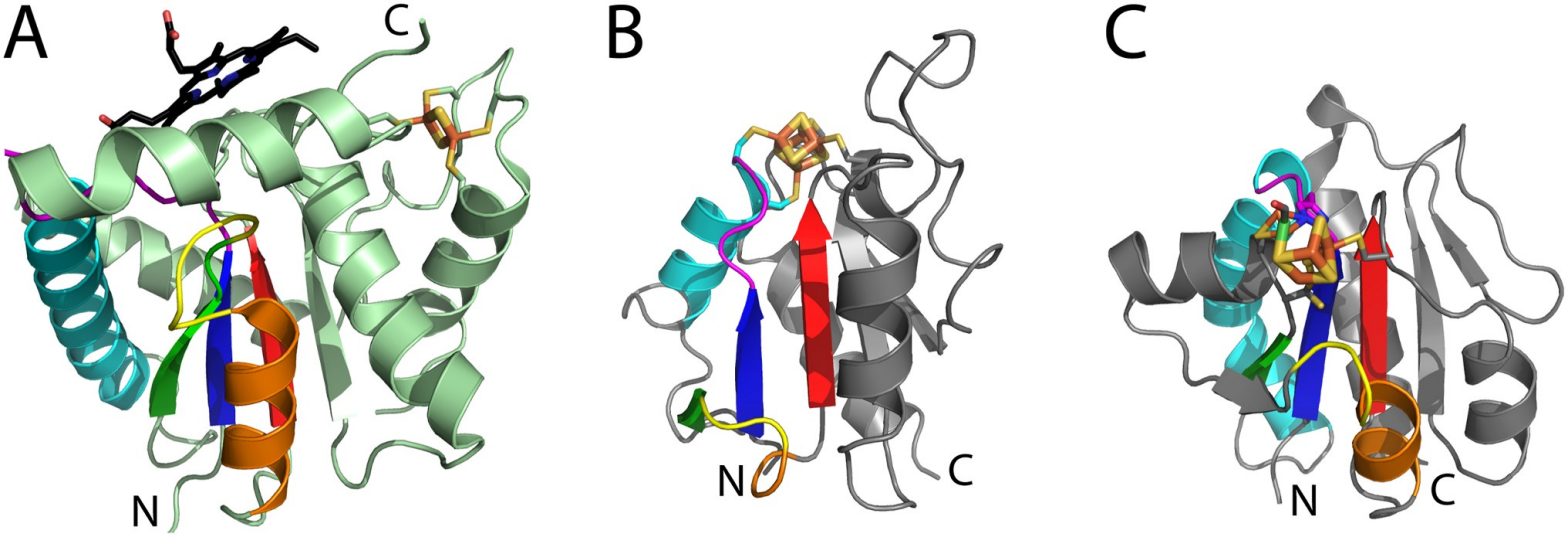
Fe-S clusters bound to RLM enzymes. RLM SSEs (colored in rainbow, with crossover loop in yellow and catalytic loop in magenta) folds bind Fe-S clusters (yellow/orange sticks, colored by element) differently. **(A)** Ferrochelatase (PDB: 2HRE) flavodoxin-like fold (light green cartoon) binds [2Fe-2S] cluster using Cys residues (sticks) from C-terminal extension to the RLM and binds protoporphyrin IX (black sticks, colored by element) using a catalytic loop insertion. **(B)** Respiratory Complex I NDUFS 7 (PDB: 5LC5, chain B) from a domain in the X-group other Rossmann-like fold with the crossover binds [4Fe-4S] using Cys residues (stick) from RLM α1 and from the C-terminal extension. **(C)** CODH/ACS (PDB: 1OAO) prismane 3^rd^ domain from the same X-group as in B (gray cartoon) binds [4Fe-4S] using residues (stick) from RLM catalytic loop, crossover, and loop C-terminal to β1.

Table 4 enumerates characteristics of iron-sulfur enzymes (EC numbers) from our data set. The smallest iron-sulfur cluster, [2Fe-2S], is the ligand for six enzymes in our data set. One example is human ferrochelatase (PDB: 2HRE, Fig 11A), essential for heme production [70]. This protein contains two flavodoxin-like domains, with Fe-S binding predominantly to the N-terminal domain through a Cys residue from the loop directly following the RLM and other Cys residues from a C-terminal helical extension to the fold [70]. The most common (83%) Fe-S binding RLM enzyme in our dataset binds [4Fe-4S], which functions mainly in oxidoreductase reactions (EC 1). An example of [4Fe-4S] binding site in RLM enzymes is dehydrogenase [ubiquinone] iron-sulfur protein 7 (NDUFS7) from mammalian respiratory complex I (EC: 1.6.5.3, 1.6.99.3) [71]. NDUFS7 uses RLM helix α1 (Fig 11B) to bind [4Fe-4S] and transport electrons via the Fe-S cluster.

**Table 4:** Characteristics of Fe-S cluster RLM enzymes.

EC	PDB repr.	ECOD H-gr	ECOD F-gr	Protein name	Cluster	Cluster binding
1.5.8.2	2tmdA	2003.1	2003.1.3.9	Trimethylamine dehydrogenase	[4Fe-4S]	N-terminal deteriorated α-helix
1.3.1.34	1ps9A		2003.1.3.40	2,4-Dienoyl CoA Reductase	[4Fe-4S]	N-terminal deteriorated α-helix
1.20.9.1	1g8kA	2003.2	2003.2.1.25	Arsenite oxidase	[3Fe-4S]	Contains two RLM domains. FeS cluster interacts with N-terminal part of α-helix α1 of only one RLM domain, binds with Cys of non-RLM domain
4.2.1.112	2e7zA			Acetylene hydratase	[4Fe-4S]	
1.17.1.9 1.17.99.7	2iv2X			Formate dehydrogenase H	[4Fe-4S]	
1.9.6.1	2jimA			Nitrate reductase	[4Fe-4S]	
1.7.5.1	1q16A			Nitrate Reductase A	[4Fe-4S]	
1.17.5.3	1kqgA			Formate dehydrogenase-N	[4Fe-4S]	
1.3.7.7	2ynmA,B	2004.1	2004.1.1.59	Light-independent protochlorophyllide reductase	[4Fe-4S]	Cluster binds between two P-loop domains from chains A and B using crossover α-helix α2 and loop after β3
1.18.6.1	1xd8A,B			Nitrogenase Fe	[4Fe-4S]	
3.6.4.12	4a15A		2004.1.1.74	ATP-dependent 5'-3' DNA helicase	[4Fe-4S]	Cluster binds to helical insertion in RLM domain
1.3.7.7	2ynmC,D	2007.1	2007.1.14.0	Light-independent protochlorophyllide reductase	[4Fe-4S]	Binds to α-helix α1 and N-terminal extension of RLM
1.18.6.1	4xpiA		2007.1.14.6	Nitrogenase Fe	[8Fe-7S]	Contains three RLM domains. Cluster binds to N-terminal domain with α-helix α1
1.18.6.1	4xpiA		2007.1.14.6	Nitrogenase Fe	[7Fe-Mo-9S]	Contains three RLM domains. Cluster binds to catalytic loop, α-helix α1 and C-terminal extension
2.5.1.108	3lzdA		2007.1.16.3	Dph2 (diphthamide syntase)	[4Fe-4S]	Contains three RLM domains. Cluster binds to crossover loop and loop after β3
4.99.1.1	2hrcA		2007.1.14.4	Ferrochelatase	[2Fe-2S]	Contains two RLM domains. Cluster binds in asymmetric mode with α-helix α1 and C-terminal α-helical extension
4.2.1.157	3o3mA		2007.1.14.5	(R)-2-Hydroxyisocaproyl-CoA Dehydratase	[4Fe-4S]	Cluster binds between two domains using α-helix α2 and loop after β3
4.2.1.3	6acnA	2111.40	2111.40.1.1	aconitase [citrate(isocitrate) hydro-lyase]	[4Fe-4S]	Cluster binds with crossover loop
1.12.7.2	4xdcA	2111.56	2111.56.1.1	[FeFe]-hydrogenase CpI	[4Fe-4S]	Cluster binds with insertion part in crossover and loop after β3
2.4.2.14	1ao0A	2111.73	2111.73.1.1	Glutamine phosphoribosylpyrophosphate amidotransferase	[4Fe-4S]	Cluster binds by N/C-terminal extensions to RLM
1.2.7.10	5c4iF	2111.75	2111.75.1.5	Thiamine pyrophosphate dependent oxalate oxidoreductase	[4Fe-4S]	Cluster binds by side of α-helix α1 and N-terminal extension
1.2.7.1	2c42A			Pyruvate-ferredoxin oxidoreductase	[4Fe-4S]	
1.2.7.4	2z8yA	2111.87	2111.87.1.4	Carbon monoxide dehydrogenase/acetyl-CoA synthase	[Ni-Fe-S]	Cluster binds between two RLM domains using catalytic loop, crossover and loop following RLM
1.7.99.1	1gn9A			Hybrid Cluster Protein	[Fe-O-S]	
1.12.99.6	4iucS	2111.89	2111.89.1.1	[NiFe]-hydrogenase	[3Fe-4S]	Cluster binds with catalytic loop, α-helix α1 and C-terminal extension
1.12.99.6	4kl8S				[4Fe-4S]	
1.12.2.1	1uboS				[4Fe-4S]	
1.6.5.3 1.6.99.3	5lc5B			NADH dehydrogenase	[4Fe-4S]	
2.5.1.72	4p3xA	2111.101	2111.101.1.1	Quinolinate synthase (NadA)	[4Fe-4S]	Cluster bind between three RLM domains with C/N-terminal extensions
1.17.7.4	3zglA	3564.1	3564.1.1.1	4-hydroxy-3-methylbut-2-enyl diphosphate reductase (IspH/LytB)	[4Fe-4S]	Cluster bind between three RLM domains with N/C-terminal extensions

Fe-S enzymes are thought to have appeared very early during the evolution of life [30, 44, 72]. Numerous Fe-S reaction mechanisms contributed to biochemical pathways from the last universal common ancestor of all cells (LUCA), including the Wood-Ljungdahl (WL) or reductive acetyl-coenzyme A pathway [30]. An RLM Fe-S bifuctional enzyme acetyl-CoA synthase carbon monoxide dehydrogenase/acetyl-CoA synthase (CODH/ACS), used by some bacteria and archaea today, acts as a component of this ancient WL pathway. The CODH reaction mechanism is responsible for reduction of CO_2_ to CO with the help of electrons from a Ni-Fe-S cofactor. The CODH catalytic center is located at the interface of two RLM domains that likely arose from a duplication (classified in the same “Rossmann-like domain in prismane-like proteins” H-group). The N-terminal RLM domain coordinates the cofactor from the catalytic, crossover, and C-terminal loops (Fig 11C), while the C-terminal RLM contributes residues from the catalytic and crossover loops. Subsequent condensation of the carbonyl group (CO) with a methyl group by another prismane-like RLM domain in ACS produces a metal-bound acetyl group, which is released from the enzyme via thiolysis, yielding a thioester product [73]. Thioesters are energy-rich and highly reactive compounds that can be used as a source of energy, like ATP, and are proposed to be essential in early metabolism [44, 74]. Thus, Fe-S binding RLM domains from prismane-like proteins likely evolved in the early stages of biochemical evolution.

### RLM domains bind nucleic acids

The second largest class of pathways, which includes RLM protein-associated 21 EC numbers, is “Genetic Information Processing”. Each of these 21 enzymes can only be observed in a single pathway–“Aminoacyl-tRNA biosynthesis”, which contains 31 unique EC numbers (KEGG Pathway ID: map00970). Consequently, 68% (21 out of 31) of enzymes in “Aminoacyl-tRNA biosynthesis” contain an RLM. 76% (16 out of 21) of these enzymes are ligases (EC: 6.X.X.X) including 15 aminoacyl-tRNA synthetases (aaRSs).These proteins belong to an ancient group and are present in all living organisms. They catalyze the attachment of amino acids to tRNA [75]. They belong to two ECOD homology H-groups whose structures converged to include the RLM: HUP domains (2005.1, class I aaRSs) and Anticodon-binding domain of Class II aaRS (2111.10, class II aaRSs). Distribution of these proteins into ECOD homology groups is in agreement with their previous classification [76]. Thus, aaRSs from class I (LeuRS, MetRS, TrpRS, etc.) belong to the 2005.1 H-group, and aaRSs from class II (GlyRS, ProRS, ThrRS, etc.) belong to the 2111.10 H-group. The final ligase is glutaminyl-tRNA synthase (EC: 6.3.5.7), which belongs to the amidase signature (AS) enzymes homology group (2111.49) and takes part in Gln and Asn metabolism [77]. The RLM domain of glutaminyl-tRNA synthase consists of a middle β-sheet, formed by 11 antiparallel β-strands, flanked by 11 α-helices. The RLM does not interact with tRNA but it does take part in binding Gln or Asn by the crossover loop (S5D Fig).

The RLM HUP domain defines the core of the aaRSs in class I. The active site of these proteins are characterized as two consensus sequence motifs: KMSKS (Lys-Met-Ser-Lys-Ser) and HIGH (His-Ile-Gly-His) [78]. The HIGH motif locates at the N-terminal end of the RLM α1, while the KMSKS (S5E Fig colored by purple) motif is located outside of the RLM. Eleven aaRSs from our data set belong to class I. Each of these enzymes binds ATP and its analogs in a similar way: the adenine ring binds with an angle of about 30 degrees relative to the middle β-sheet plane and interacts with both motifs. The phosphate group binds the HIGH (S5E Fig colored by yellow) motif, and catalytic loop. Class II aaRSs contain the RLM as a C-terminal, non-enzymatic domain of the protein. The class II RLM binds the anticodon stem of tRNA using residues from the RLM α2 and the β-sheet (S5F Fig). Thus, the mode of class II aaRS RLM tRNA binding differs from that of the class I aaRS catalytic RLM domain, whose interaction is mainly through the loops.

Other RLM proteins in the Aminoacyl-tRNA biosynthesis pathway function as transferases (EC: 2.X.X.X, 5 out of 21), and they belong to three ECOD homology groups: PLP-dependent transferases (2111.77), formyltransferases (2111.71) and P-loop domains-related (2004.1).

The PLP-dependent transferases (EC: 2.9.1.1, ECOD: e3w1kA4; EC: 2.9.1.2, ECOD: e4zdoA1; EC: 2.5.1.73, ECOD: e2e7iA2) bind PLP using residues of the RLM catalytic loop and α1. tRNA binds between units of the oligomer and does not interact with the RLM (S5A Fig). Alternately, formyltransferase (EC: 2.1.2.9, ECOD: e2fmtA2) binds tRNA using a C-terminal non-RLM domain, as well as the catalytic, crossover and C-terminal loop after β3 of RLM (S5B Fig). Finally, the P-loop (EC: 2.7.1.164, ECOD: e3am1A1) binds ATP using a P-loop motif in the RLM catalytic loop and α1. The 3′ end of tRNA binds to the RLM domain, interacting with the crossover loop and α2 (S5C Fig).

### RLM enzymes inhibited by methotrexate reveal its side effects significance

**Fig 12:**
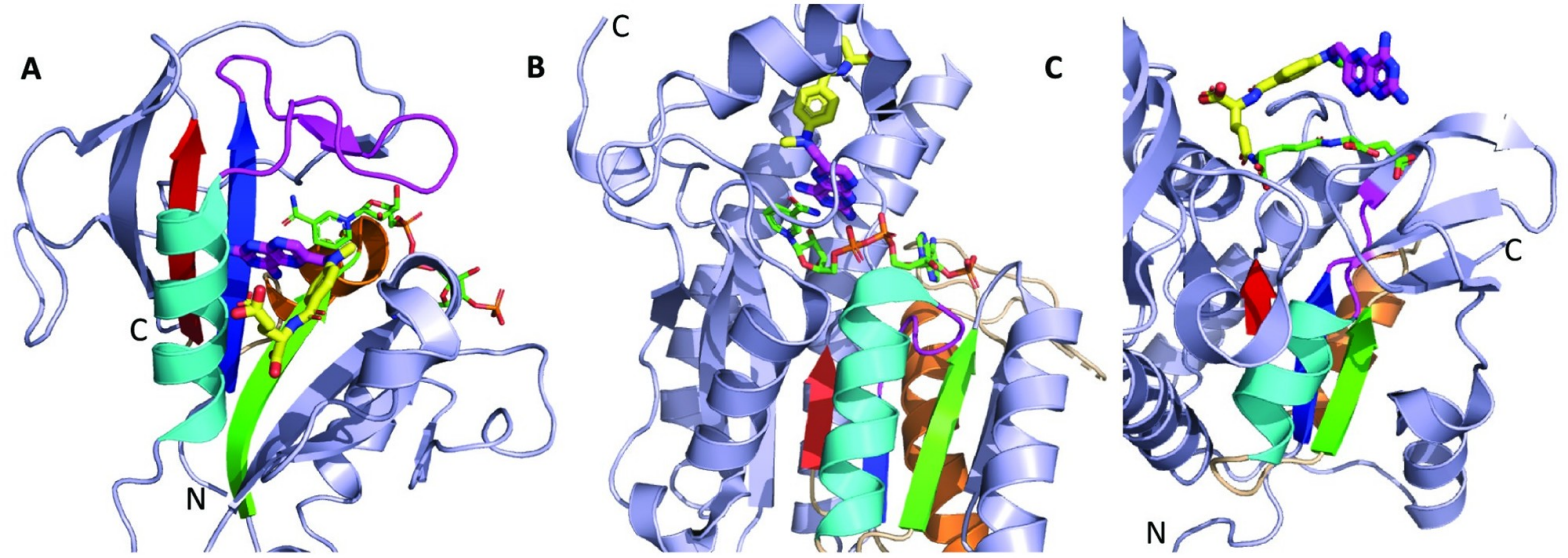
ECOD homology groups reveal different methotrexate binding modes in RLM enzymes. Domains with RLM SSEs (colored in rainbow, with crossover loop in yellow and catalytic loop in magenta) bind methotrexate (shown in thick sticks; pteridine ring Cα atoms are colored by magenta, rest by yellow) differently. **(A)** DHFR (PDB: 1U72, EC: 1.5.1.3, ECOD H-gr: 2111.5) binds methotrexate and NADH (green sticks, colored by element). **(B)** Pteridine reductase (PDB: 1E7W, EC: 1.5.1.33, ECOD: 2003.1) binds methotrexate and NADH (sticks, colored by element). **(C)** Gamma-glutamyl hydrolase (PDB: 4L8W, EC: 3.4.19.9, ECOD: 2007.1) binds methotrexate and glutamic acid (sticks, colored by element).

We observe several cases where the same drug is bound to multiple enzymes with different topologies. These enzymes are interesting cases for polypharmacology, which suggests that clinical effects of drugs are often observed because of the interaction of single or multiple drugs with multiple targets, not a specific “key-lock” mechanism with a single target. One such example is methotrexate (PDB ligand: MTX, DrugBank ID: DB00563), an inhibitor of tetrahydrofolate dehydrogenase. Due to the important role of folate in the synthesis of DNA, RNA, thymidilates, and proteins, methotrexate is used as treatment for various cancers. Currently, 55 protein structures (RLM and non-RLM) deposited in the PDB bind methotrexate. 46 of these 55 proteins are enzymes with six different catalytic activities, including dihydrofolate reductases (67%, EC: 1.5.1.3), thymidylate synthases (EC: 2.1.1.45), pteridine reductases (EC: 1.5.1.33), folate conjugases (EC: 3.4.19.9), S-methyltransferase (EC: 2.1.1.14), and a bi-functional enzyme (EC: 3.5.4.9;1.5.1.5). Most (87%, 48 out of 55) of these proteins contain an RLM and belong to three different ECOD homology groups: 2111.5 (41 out of 48 RLM), 2003.1 (5 out of 48) and 2007.1 (3 out of 48). Structures of the RLM domains reveal three different binding sites of this drug, which are distinctive for each particular homology group (Fig 12A–12C).

DrugBank [79] contains one target of methotrexate with confirmed pharmacological action: human dihydrofolate reductase (DHFR, UniProt ID: P00374, EC: 1.5.1.3). DHFR catalyzes the reduction of folic acid to dihydrofolic acid and tetrahydrofolic acid, an essential cofactor in the biosynthesis of thymidylate, purines, and glycine. Methotrexate is an analog of folic acid and binds to the active site of DHFR (Fig 12A), resulting in the death of exposed cells [80]. Residues from all RLM structural elements take part in drug binding. The pteridine ring of the drug interacts with the nicotinamide ring of NAD cofactor, as well as with all RLM structural elements. DHFR belongs to the ECOD Dihydrofolate reductases homology group (ECOD: 2111.5). This homology group contains two families: “DHFR_1” containing dihydrofolate reductases (ECOD: 2111.5.1.1) and “RibD_C” containing the C-terminal domains of RibD proteins (EC: 1.1.1.193), which take part in riboflavin biosynthesis (ECOD: 2111.5.1.2). The structural features of these two families are similar (Dali Z-score 13.7 [81]), including the methotrexate binding pocket. The RibD pocket binds 5-amino-6-ribosylamino-2,4(1H,3H)-pyrimidinedione 5-phosphate (PDB ligand ID: AI9, PDB: 4G3M) [82] using a similar orientation as methotrexate in DHFR. Since methotrexate appears to be a strong inhibiting agents capable of binding multiple non-homologous protein target, these data might suggest that it could also inhibit RibD riboflavin biosynthesis.

A second RLM methotrexate-binding mode is illustrated by *Leishmania major* pteridine reductase (PTR). PTR catalyzes the reduction of 5,6,7,8-tetrahydrobiopterin (THBP) to biopterin and it is a component of pterin and folate metabolism [83]. PTR is essential for growth of trypanosomatid protozoans like *Leishmania* parasites, thus the PTR inhibitor methotrexate was tested as a potential drug [84]. PTR belongs to one of the largest RLM “Rossmann-related” homology groups (ECOD: 2003.1). In this case, the pteridine ring of the drug is also located adjacent to the nicotinamide ring of NADH and interacts with the N-terminal part of the α1 (Fig 12B). 11beta-hydroxysteroid dehydrogenase type 1 (11beta-HSD1) belongs to the same homology group as PTR. The 11beta-HSD1 structure resembles PTR (PDB: 1Y5R, Dali Z-score 24.8), but exhibits different enzymatic function (EC: 1.1.1.146). 11beta-HSD1 catalyzes the conversion of 11-dehydrocorticosterone to its active form corticosterone in rodents (or cortisone to cortisol in humans) [85]. Different diseases, such as type II diabetes, dyslipidemia, and obesity, are induced by glucocorticoid excess. The therapeutic potential of 11-HSD1 inhibition has been shown previously [86]. The 11-HSD1 structure (PDB: 1Y5R) has two ligands: NADPH, which binds at the same position as NADH binds to PTR, and corticosterone (DrugBank ID: DB04652), which binds at the same pocket as methotrexate binds to PTR. The structural similarity between these two enzymes suggests a possibility that methotrexate also might bind and inhibit 11beta-HSD1.

The third example of methotrexate binding is illustrated by gamma-glutamyl hydrolase (Fig 12C). This protein is a lysosomal peptidase that catalyzes the hydrolysis of gamma-linked glutamate residues from folate derivatives such as methotrexate [87]. Gamma-glutamyl hydrolase belongs to the “GATase” family (ECOD: 2007.1.1.6), which belongs “Flavodoxin-like” homology group (ECOD: 2007.1). The β-hairpin insertion to the RLM catalytic loop interacts with the pteridine ring of the drug (Fig 12C). Methotrexate exhibits numerous side effects [88] from off-target interactions. Our data suggest that methotrexate is capable of inhibiting several RLM enzymes, for which this inhibition was previously shown. This drug also inhibits enzymes such as histone deacetylases (EC: 3.5.1.98, ECOD: 2006.1) [89], malate dehydrogenases (EC: 1.1.1.37, ECOD: 2003.1), isocitrate dehydrogenases (EC: 1.1.1.41, ECOD: 2111.4) and oxoglutarate dehydrogenases (EC: 1.2.4.2, ECOD: 2111.14 and 2111.75) [90]. Due to the tremendous diversity of RLM domains and their recurrence in nature by convergent evolution, these proteins adopt functions of all main enzymatic classes and interact with a wide range of chemical compounds. Our data showed that non-homologous RLM domains are capable of binding the same ligand with alternate binding modes, that might cause severe side effects in the case of strong inhibiting agent like methotrexate.

### Conclusions

In this work, we defined the minimal Rossmann-like structure, found proteins which contain this motif and described the common binding sites of these RLM domains and their associated ligands. The ability of these proteins to bind many types of ligands is provided by the incorporation of an RLM into a broad array of structural contexts, as well as their structural features. Being a doubly-wound three-layer sandwich, an RLM protein harbors a spacious cavity centered between the two halves of the domain, right between the first β-strand of the protein and the first β-strand after the crossover. This cavity, frequently covered by insertions and additional domains, can accommodate diverse ligands of all sizes and shapes. Detailed placement of binding sites and ligand-binding modes vary among different RLM proteins. However, we show that on average, the secondary structure elements constituting the minimal RLM are more conserved in sequence than the rest of the domain, and the RLM components mostly take part in binding ligands.

Our comprehensive analysis confirms that the most prevalent class of chemical compounds binding to RLM enzymes includes nucleotides and their analogs. We find that homologous RLM proteins tend to have similar binding sites. However, even closely related RLM enzymes can catalyze different chemical reactions using a similar overall topology and can diverge to bind different ligands albeit in the similar binding sites. Conversely, non-homologous RLM domains can converge to catalyze the same reactions or to bind the same ligand using different binding modes. Generally, it is typical for non-homologous RLM proteins to exhibit different locations of the functional site and to bind different compounds. Furthermore, functional sites of RLM proteins bind drugs, typically in place of their natural ligands. We see that strong binding agents, such as methotrexate, which is used to treat a variety of cancers, are capable of inhibiting multiple non-homologous RLM enzymes and at multiple sites. The presence of multiple methotrexate-binding sites in a number of non-homologous RLMs is relevant in the light of polypharmacology.

Being ubiquitous in nature, RLM proteins constitute nearly 40% of enzymes in metabolic pathways and are particularly overrepresented in the pathways thought to be most ancient, such as nucleotide metabolism, energy metabolism, and metabolism of amino acids. Moreover, the abundance of RLM proteins that bind a variety of iron-sulfur clusters and their involvement in the Wood-Ljungdahl metabolic pathway suggested to be used by the LUCA implies their ancient origins and importance at the early stages of evolution of life. Taken together, our data reveal that RLM represents a highly successful ligand-binding domain, which arose several times in evolution and was used by life systems since the times of LUCA.

## Materials and methods

### Identifying RLMs in ECOD domains using ProSmoS

The minimal RLM was defined as a three-layer α/β/α sandwich with the central β-sheet containing a minimum of three parallel β-strands (β1, β2, and β3 in Fig 1). We require the second element to be α-helical to maintain the α/β doubly-wound characteristic of Rossmann-like folds and to maintain the known ligand binding site. To accurately represent all known Rossmann-like crossover connections between β-strands β2 and β3, element IV includes three variations: α-helix, β-strand or linker (Fig 1B).

We used the minimal RLMs described above as queries to search against protein structures in the PDB using the ProSMoS program developed in our lab [22]. We used PALSSE [91] to generate a database of secondary-structure interaction matrices derived from ECOD domains (database version: develop214/20181017). Each matrix describes the interactions (parallel or antiparallel) and hydrogen-bonding of the PDB structure. This minimal structural consensus of RLM domains was represented as three ProSmoS query matrices. Query matrices specified the number and types of secondary structure elements in the motif under consideration, the hydrogen-bonding and parallel or anti-parallel relationships between its elements, and minimum and maximum length of the three component β-strands. All β-strands were required to be at least three amino acids in length. Out of more than 80,000 domains, which contain RLM, only 840 domains were not identified by ProSmoS. There were several reasons for this: deteriorated or missed RLM β-strands (e5da1A2), unusual element IV (e3i12C4).

Domains were considered to belong to a Rossmann-like fold when the RLM overlapped with the evolutionarily conserved structural core. Domains from each PDB structure from the ProSMoS search results were annotated using ECOD. ECOD domains are identified by an identifier (e.g. “e2cx8A1”), which incorporates the a) PDB identifier, b) a chain identifier (sometimes multicharacter), and c) a domain number. In the current work we used the following hierarchy of structural definitions: Structures/depositions from the PDB contain multiple proteins/chains which can contain one to many ECOD domains. We defined “fold” as synonymous to ECOD topology groups. All RLMs identified must overlap completely with an ECOD domain to be considered. Consequently, each identified RLM is fully contained as part of an ECOD domain and can not belong to two different domains at the same time.

To calculate average conservation in RLM (Fig 1C) we used AL2CO positional conservation index [23]. Each RLM element was divided into bins. Number of bins for each element was set based on the average length distribution of these elements in representative RLM domains at the ECOD F-group level (S4C–S4G Fig). The length of a particular element of representative domain was partitioned (if RLM element has more residues than average number of bins) or stretched (if RLM element has less residues than average number of bins) to fit the number of bins. In the similar way, the plot of conservation index versus residue number for each particular RLM element was partitioned or stretched to fit the numbers of bins. Conservation index value for a particular bin was calculated as the value of the function described plot of conservation index versus residue number in the center of this bin. For each bin, all values were summarized, and average values were calculated for all representatives.

### Mapping ligands to Rossmann-fold containing proteins

For each protein containing one or more ECOD domains containing a Rossmann-fold motif, we identified associated catalytic activities by the Enzyme Commission number (EC number). EC assignments were determined using annotations from the Protein Data Bank [3] for each PDB and the UniProt Knowledge Base (release 2018_10) [21] for each UniProt sequence. Proteins were considered to be multifunctional if either the PDB Data Bank or UniProt KB contained multiple EC number assignments for that particular protein. To evaluate the consistency of chemical reactions performed by ECOD families (F-groups), we examined EC assignments among all ECOD family members in each F-group. We first identified 1259 unique ECOD family groups among the RLM containing protein structures. For those structures with multiple RLM domains, we described each combined domain organization as unique (i.e. a structure with the single F-group 2007.2.5.2 is one family and one with multiple F-groups 2007.2.5.2 and 2003.1.2.285 is another family). The resulting list of families were divided into four categories according to their family member EC numbers: (i) null F-groups with no EC assignment, (ii) homogeneous F-groups with the same EC number, (iii) heterogeneous substrate F-groups with similar EC numbers (same first three digits), and (iv) heterogeneous reaction F-groups with different EC numbers.

We collected all functional ligands associated with EC numbers from RLM PDBs that were defined as “Substrate” and “Product” by the KEGG Compound database [20] and defined as “Cofactor” by UniProt KB. Each of these compounds were then classified into “Kingdoms”, “Superclasses” and “Classes” according to their chemical features using the ClassyFire taxonomy database [11]. Since not all compounds could be classified using this database, we constructed an additional group, “Generic compounds”, at the superclass level. This *ad hoc* group included compounds containing an "R" group representing a range of chemical compounds (e.g. R-Br; KEGG ID: C00720), amino acids connected to a nucleic acid (e.g. L-Lysyl-tRNA; KEGG ID: C01931), and entire proteins that take part in chemical reactions and could be considered as substrates or products (e.g. ubiquitin; KEGG ID: C00496). We manually classified CTP and its derivatives to the nucleotides superclass, since all nucleotide-like compounds are observed in this superclass: C00063, C00705, C05673, C05674, C05822.

To gain insight into the structural characteristics of Rossmann-like active sites, we identified all ligands present in structures contacting RLM domains. All ligands found within a distance of 4Å of a domain containing the RLM were collected. We mapped these PDB ligands to the KEGG Compound database using SIMCOMP search tool [92] using the Simplified Molecular-Input Line-Entry System (SMILES) [93] formula for each ligand as the input. SMILES formula for each ligand was derived from ligand web page in Protein Data Bank (S2 Table). We defined these collected PDB ligands as biologically significant if they correspond to their EC number substrate, the product from the KEGG Compound database, or a cofactor from the UniProt KB. Otherwise, it was considered as ligand of uncertain significance.

Superpositions for Fig 6 were obtained using following algorithm. First, we chose representative domains from each H-group that bind ligands of particular superclass. Second, from each PDB structure of representative domains we cut out the RLMs using ProSmoS search results. Using TMalign [94] we aligned all RLMs inside one H-group to one representative, chosen to be shown in the Fig 6, and obtained translation and rotation matrices for each case. Coordinates of all representative PDB structures were modified using these matrices by multiplying to rotation matrix values and adding translation vector values. Rotated domains were written as single file and visualized using PyMOL (The PyMOL Molecular Graphics System, Version 2.0 Schrödinger, LLC). Each atom of all ligands was shown as spheres, which were colored according to compounds superclass color in legend to the Fig 6A. To verify the quality of obtained superpositions, we visualized superpositions of RLMs only for each H-group (S6 Fig).

The DrugBank database [79] contains detailed drug data as well as comprehensive drug target information. We obtained drug targets of methotrexate from this database using its DrugBank identification number (DB00563), which we retrieved from ligands section of Protein Data Bank (http://www.rcsb.org/ligand/MTX).

### Inorganic cofactors analysis

For each PDB in our dataset we collected information about inorganic cofactors from UniProt KB. In the case of metal cations, most proteins have specific ions defined as cofactors, but some cases have broader definitions (i.e. “a divalent metal cation”). If UniProt KB specified that an enzyme had “a divalent metal cation” as a cofactor, we checked for any specific cations fitting this criterion in the structure. In our data set we observed the following range of metal cofactors (not necessarily present in structures): Mg^2+^, Zn^2+^, Mn^2+^, Co^2+^, Ca^2+^, Ni^2+^, Fe^2+^, Cu^2+^, Cd^2+^, Na^+^, K^+^, Fe^3+^. The second group of cofactors is the iron-sulfur clusters, which are divided into seven categories according to their composition. Two types of clusters ([Fe-O-S] and [Fe-Ni-S]) are defined by literature as hybrid clusters. We observed several variations of these hybrid clusters in our data set, however we merged them into two types regardless of composition. We mapped all iron-sulfur cofactors defined by UniProt KB to EC numbers and the family groups to which they bind in our data set.

### Pathway mapping

To map EC numbers from our dataset to pathways described in the KEGG Pathway database, we used the KEGG Mapper tool searching “map” pathways. RLM enzymes were mapped to the Reference Metabolic Pathway (KEGG Pathway ID: map01100) as black lines using the “User data mapping” tool.

In total, we mapped 18,824 PDB structures from our data set to 1472 unique EC numbers. 6% (94 out of 1472) of obtained EC numbers contained unknown digits–dashes. Such EC numbers could not be mapped to any pathway and were removed from the mapping set. We mapped the remaining 1378 EC numbers, which were assigned to the Rossmann-containing PDB structures, to all known pathways from KEGG Pathway database using KEGG Mapper tool against “map” pathways. The KEGG Pathway database contains seven pathway groups in its top hierarchal level, which we define as “major pathways groups”. The first group (“Metabolism” in KEGG) was defined as “global metabolism”. Each major pathways group contains several smaller groups, which we define as “classes”.

In order to calculate over- and underrepresentation of RLMs in biological pathways, we mapped RLM EC numbers to each KEGG pathways class (and pathways within that class) using the KEGG Mapper tool. The result of this mapping was a list of non-redundant EC numbers for each pathways class. Similarly, for each major pathway groups we generate a list of non-redundant EC numbers mapped to that group. For example, 939 RLM EC numbers were mapped to the global metabolism major pathways group, whereas 21 RLM EC numbers were mapped to the genetic information processing major pathway group, as discussed above. The collection of all EC numbers mapped to a particular pathway was derived from the “Pathway entry” web page in the KEGG Pathway database. The collection of all (non-RLM and RLM) EC numbers observed in a particular pathways class is a non-redundant list of all EC numbers mapped to all pathways of this class. Similarly, the collection of all (non-RLM and RLM) EC numbers mapped to a particular major pathways group is generated by collected the mapped pathways from each pathways class and removing redundant EC numbers. Over- and underrepresentation of RLM enzymes in classes of the global metabolism major group (Fig 2A) were calculated as ratio of observed and expected frequencies. The observed frequency in each pathways class of global metabolism was calculated as a ratio of the total number of the RLM EC numbers in a particular class over the sum of RLM EC numbers in the global metabolism major group. The expected frequency in each pathways class of global metabolism was calculated as ratio of total (non-RLM and RLM) EC numbers found in each particular class to the total amount of EC numbers in the global metabolism major group. The significance of this result was established using 2x2 contingency tables for Fisher’s exact test. For this test, we classified each enzyme as either RLM, or non-RLM. We checked the number of RLM and non-RLM enzymes in each pathways class versus the total number of RLM and non-RLM EC numbers in global metabolism major group.

## Supporting information

S1 Appendix Detailed description of RLE in TIM barrels. (DOCX)

S2 Appendix Detailed description of top three ECOD family groups with largest number of unique EC numbers from Table 1. (DOCX)

S3 Appendix Description of major binding modes of the top 10 most populated RLM H-groups from Table 2. (DOCX)

S4 Appendix Description of 5’-deoxyribonucleosides ligands class binding modes for major H-groups. (DOCX)

S1 Fig ECOD RLM X-groups statistics. (PNG)

S2 Fig RLE adopt open conformation of lid domain in EDTA monooxygenase (PDB: 5DQP, chain A) The RLE is colored by rainbow. Polyethylene glycol is represented by sticks and colored by element. Moving part is colored in light pink.
(PNG)

S3 Fig Standard errors of conservation index for each bin of all RLM elements (from Fig 1C): (A) RLM element I (β1), (B) RLM element II (⍺1), (C) RLM element III (β2), (D) RLM element IV (⍺2), (E) RLM element V (β3). (PNG)

S4 Fig Distribution of AL2CO conservation index values inside and outside of RLM. **(A)** Normal scale. **(B)** Logarithmic scale. **(C-G)** Length distribution of all RLM elements among representative domains.
(PNG)

S5 Fig RLM enzymes bind RNA. **(A)** PLP-dependent transferase (EC: 2.9.1.2, ECOD: e4zdoA1) binds tRNA and PLP (shown as sticks, colored by element). **(B)** Formyltransferase (EC: 2.1.2.9, ECOD: e2fmtA2) binds tRNA and N-formylmethionine (shown as sticks, colored by element). **(C)** O-Phosphoseryl-tRNA kinase (EC: 2.7.1.164, ECOD: e3am1A1) binds tRNA, ATP (shown as sticks, colored by element) and Mg (green sphere). **(D)** Glutaminyl-tRNA synthase RLM domain does not interact with tRNA. **(E)** Methionyl-tRNA synthetase class I (EC: 6.1.1.10, ECOD: e2ct8A2) binds tRNA and 5'-O-[(L-Methionyl)-sulphamoyl]adenosine (shown as sticks, colored by element). HIGH motif colored in yellow, KMSKS motif colored in purple. **(F)** Histidinyl-tRNA synthetase (EC: 6.1.1.21, ECOD: e4rdxA2) binds tRNA. (A-F) RLM colored in rainbow.
(PNG)

S6 Fig Superpositions of biggest 10 H-groups RLMs, that correspond to H-groups in Fig 6. (PNG)

S7 Fig RLM enzymes reactome. RLM protein EC numbers (black arrows) mapped to KEGG reference metabolic pathways function in all major categories: glycam biosynthesis and metabolism (light blue), lipid metabolism (green), metabolism of terpines and polyketides (lime green), xenobiotics biodegredation and metabolism (salmon), carbohydrate metabolism (blue), amino acid metabolism (orange), energy metabolism (purple), nucleotide metabolism (red), metabolism of cofactors and vitamins (pink), metabolism of other amino acids (dark orange), and biosynthesis of other secondary metabolites (magenta).
(PNG)

S8 Fig Ligands from RLM catalyzed reactions. Combined KEGG compounds and UniProt cofactors (colored and classified according to ClassyFire superclass in legend) by assigned EC reaction count (Y-axis, cutoff at 100, with total number for EC>100 indicated) are distributed across ECOD Homology groups (X-axis).
(PNG)

S1 Table Distribution of all RLM-containing domains across SCOP, CATH and ECOD classifications. (XLSX)

S2 Table PDB ligands SMILES formulas. First column contains three-letters PDB IDs of each ligand derived from Protein Data Bank. Second column contains SMILES formulas that correspond to particular ligand.
(XLSX)

S3 Table ECOD X-groups in “α/β three-layered sandwiches” architecture group that that either contain RLM or not. *Classification of some of ECOD X-groups changed since time of initial paper submission.
(DOCX)

S4 Table Data set of RLM containing PDBs. Each PDB mapped to UniProt KB, ECOD database, EC number (if available). Substrate and Product information was retrieved from KEGG, Modified residues, Nucleic acid contact and Polysaccharide contact—from Protein Data Bank.
(XLSX)

S5 Table Classification of ligands associated with RLM EC numbers based on ClassyFire database. (XLSX)
