# Supplementary material for: Functional analysis of Rossmann-like domains reveals convergent evolution of topology and reaction pathways
Source: PLoS Comput Biol. 2019 Dec 23;15(12):e1007569. doi: 10.1371/journal.pcbi.1007569 (PMC6957218; doi:10.1371/journal.pcbi.1007569)
Supplement: S1 Appendix — (DOCX) [file pcbi.1007569.s001.docx]

**Detailed description of RLE in TIM barrels.**

In our data set, we observed proteins with RLM SSEs that cannot be linked to other RLM domains using sequence or structure similarity. These distinct domains might not have originated from a common Rossmann-like ancestor. In some cases, the RLM does not belong to the conserved structural core and is instead acquired as recent decoration. We define these cases as proteins that contain a minimal Rossmann-like equivalent (RLE). Only a single H-group contains an RLE: TIM barrel homology group (ECOD: 2002.1). TIM barrel domain structures are typically composed of eight repeats of a β-strand and an α-helix and are present in approximately 10% of all enzyme structures [1]. Many TIM barrel enzymes are thought to have evolved either through gene duplication or fusion of a half-barrel [2] and might be very distant homologous with flovodoxin-like fold, that contains RLM [3]. Among the variety of TIM barrel domains, only the bacterial luciferase family (ECOD: 2002.1.1.22) contains an RLE as an insertion to the core 8 β/α unit repeat (Fig S1). The unique luciferase RLE strand β1 is longer than other strands in the barrel, and the RLE is formed by an insertion between the C-terminal end of strand β1 and the N-terminal end of helix α2 (Fig S1 – light pink region and green β-strand). The length of this insertion varies within the family and consists of several α-helices and a small β-strand forming the RLE β1 strand that does not take part in the central eight-stranded β-barrel. Jun *et al.,* defined this insertion for the luciferase family member EDTA monooxygenase (PDB: 5DQP) as the “lid domain” [4]. Lid domains of this structure adopt two conformations: the open form of chain A has a *trans* conformation and closed form of chain B has a *cis* conformation [4]. Adopting the *cis* conformation, by moving the light pink region, the lid domain seals the active site upon binding its substrate [4]. Thus, the RLE is formed by insertion to the conserved core of the TIM-barrel domain and, being the basis of lid domain movement, is a functionally important structural feature of this family group.

**References**

1. Goldman AD, Beatty JT, Landweber LF. The TIM barrel architecture facilitated the early evolution of protein-mediated metabolism. J Mol Evol. 2016 Jan 1;82(1):17-26.
2. Lang D, Thoma R, Henn-Sax M, Sterner R, Wilmanns M. Structural evidence for evolution of the β/α barrel scaffold by gene duplication and fusion. Science. 2000 Sep 1;289(5484):1546-50.
3. Farías-Rico JA, Schmidt S, Höcker B. Evolutionary relationship of two ancient protein superfolds. Nature chemical biology. 2014 Sep;10(9):710.
4. Jun SY, Lewis KM, Youn B, Xun L, Kang C. Structural and biochemical characterization of EDTA monooxygenase and its physical interaction with a partner flavin reductase. Mol Microbiol. 2016 Jun;100(6):989-1003.
