# Supplementary material for: Functional analysis of Rossmann-like domains reveals convergent evolution of topology and reaction pathways
Source: PLoS Comput Biol. 2019 Dec 23;15(12):e1007569. doi: 10.1371/journal.pcbi.1007569 (PMC6957218; doi:10.1371/journal.pcbi.1007569)
Supplement: S2 Appendix — (DOCX) [file pcbi.1007569.s002.docx]

**Detailed description of top three ECOD family groups with largest number of unique EC numbers from Table 1.**

The ECOD F-group enoyl-(Acyl carrier protein) reductase (ECOD: 2003.1.1.417) contains the largest number of unique EC numbers (51), followed by aminotransferase class V (ECOD: 2111.77.1.72, 46 EC numbers) and alpha/beta hydrolase (ECOD: 2111.78.1.7, 40 EC numbers). The enoyl-acyl carrier protein reductases belong to the classic Rossmann-like X-group (ECOD: 2003) and include mainly homogeneous oxidoreductase reactions acting on the CH-OH group of donors, using NAD+ or NADP+ bound to the RLM as acceptors (EC 1.1.1), but with 41 different substrates. This group of proteins also use RLM-bound NAD(P) to catalyze five homogeneous oxidoreductase reactions acting on CH-CH groups (EC 1.3.1.X) and one that acts on CH-NH groups (EC 1.5.1.33). Thus, this enoyl-acyl carrier protein reductase family catalyzes heterogeneous reactions mediated by RLM bound NAD(P). The catalytic heterogeneity stems from binding alternate substrates using SSEs C-terminal to the RLM. Other diverse functional groups, such as the aminotransferase and alpha/beta hydrolase groups, catalyze heterogeneous reactions from four classes: including oxidoreductase, transferase, hydrolase, and lyase reactions. The aminotransferases function mainly as transferases (31 reactions) using a PLP cofactor covalently attached to a lysine residue located C-terminal to the RLM and the pyridoxal ring interacting with a conserved residue from the typical RLM active site loop. The alpha/beta hydrolases function mainly as hydrolases (28 reactions). The chemical promiscuity of this superfamily has been outlined elsewhere [1].

**References**

1. Lenfant N, Hotelier T, Bourne Y, Marchot P, Chatonnet A. Proteins with an alpha/beta hydrolase fold: relationships between subfamilies in an ever-growing superfamily. Chem Biol Interact. 2013 Mar 25;203(1):266-8.
