# Supplementary material for: Functional analysis of Rossmann-like domains reveals convergent evolution of topology and reaction pathways
Source: PLoS Comput Biol. 2019 Dec 23;15(12):e1007569. doi: 10.1371/journal.pcbi.1007569 (PMC6957218; doi:10.1371/journal.pcbi.1007569)
Supplement: S3 Appendix — (DOCX) [file pcbi.1007569.s003.docx]

**Description of major binding modes of the top 10 most populated RLM H-groups from Table 2.**

The largest X-group in our dataset (Rossmann-like; ECOD: 2003) includes two H-groups, Rossmann-related (ECOD: 2003.1) and Ribokinase-like (ECOD: 2003.6), that have different binding sites (Fig 6B). The biggest Rossmann-related ligands cluster corresponds to nucleotides and their analogs (light pink spheres, Figure 6B left panel), which has been discussed previously [1, 2]. Briefly, the first RLM helix cap binds phosphate and an Asp/Glu motif at the C-terminal end of β2 RLM strand, binds ribose from various cofactors including S-adenosylmethionine (SAM), NAD and FAD. The Asp/Glu motif is thought to reflect divergence of a common RLM ancestor [2]. A second cluster of heterogeneous Rossmann-related ligands is located adjacent to the nucleotides cluster, interacting with the loops and α-helices following β3. Alternately, the ribokinase-like homology group (ECOD: 2003.6) binds nucleotides superclass ligands (mainly ATP and ADP, pink Fig. 6B right panel) in a binding site C-terminal to the RLM that is adjacent to the typical Rossmann-like site. Ribokinase superfamily members bind two additional superclasses of ligands classified as organoheterocyclic compounds (for example PLP, dark green, Fig 6B right panel) and as organic oxygen compounds (for example GLC sugar, orange, Fig 6B right panel) in an overlapping cloud that is adjacent to the nucleotide site. This cloud binds to the catalytic loop, α1 and the loop after β3 and represents the substrate (PLP or sugar) that gets phosphorylated by ADP/ATP in the adjacent nucleotide binding site.

Comparison of the ligand distribution in two flavodoxin-like H-groups (ECOD: 2007.1 and 2007.2) also reveals binding site differences. The class I glutamine amidotransferase-like H-group (ECOD: 2007.1) has two major heterogeneous ligand clusters: nucleotides and organoheterocyclic compounds (Fig 6C, left picture). Nucleotide ligands (e.g. NAD+) interact with RLM strand β3 and the following loop. NAD-binding flavodoxin-like domains are generally components of multidomain enzymes that are fused to traditional NAD(P) Rossmann-related domains, where the NAD binding site is formed by multiple domain interactions. A second nucleotide binding site, which illustrates binding of ATP and its derivatives, is formed by residues of the RLM catalytic and crossover loops and the α-helix following strand β3. The second cluster is represented by heme and cobalamin (organoheterocyclic compounds), which function as substrates and interact with the catalytic loop, α1 and loop following β3. The flavoproteins/phosphotyrosine protein phosphatases-like H-group (ECOD: 2007.2) also contains a homogeneous cluster of nucleotide-like compounds represented by FAD and its derivative cofactors (Fig 6C, right picture, light pink spheres). These ligands interact with RLM catalytic loop, α1, β3 and non-RLM elements at the C-terminal part of the domain. Several representatives from this H-group bind drugs (e.g. primaquine and chloroquine) which belong to the organoheterocyclic compounds superclass (dark green spheres). These compounds interact with C-terminal elements of the domain and are located above the nucleotide cluster.

Two of the most populated H-groups from the “other Rossmann-like structures with the crossover” X-group (ECOD: 2111) include nucleotide-diphospho-sugar transferases (ECOD: 2111.6) and PLP-dependent transferases (ECOD: 2111.77). Nucleotide-diphospho-sugar transferases include a nucleotides cluster (Fig 6D left panel, pink spheres) consisting mainly of UDP, GDP, and their analogs that interacts with all RLM elements except β3. In contrast to the above described Rossmann-like X-group domains (Figure 6A, left panel), which use the α1 cap to bind nucleotide phosphate, the nucleotide-diphospho-sugar transferases bind nucleotide phosphate using residue side chains from α1 and loops C-terminal to the RLM. These enzymes bind their sugar substrates (Figure 6D left panel, orange spheres) in a cloud adjacent to the nucleotide using elements C-terminal to the RLM. In contrast, PLP-dependent transferases (ECOD: 2111.77) do not bind nucleotides but instead utilize the organoheterocyclic compound PLP cofactor (dark green, Fig 6D, right picture). PLP forms a Schiff-base with a lysine from a loop C-terminal to the RLM and binds at an RLM dimer interface. Elements from the RLM (the catalytic loop and the C-terminus of β3) bind the pyridoxal ring, whereas an α-helix cap and loops C-terminal to the RLM bind the PLP phosphate. A second ligand cluster composed of mainly organic acids and derivatives (violet spheres, Fig 6D, right picture) binds close to the PLP position and represents various substrates and products of the reaction. These ligands interact with RLM α1 but primarily bind to the non-RLM C-terminal domain. Several cases in this H-group illustrate binding of succinyl-Coenzyme A, which belongs to the lipids and lipid-like molecules superclass (cyan spheres). For example, in the protein 5-aminolevulinate synthase (PDB: 2BWO) this extended compound binds at a dimer interface. The adenine ring interacts with β1, the catalytic loop, α1 and β2, while the CoA ribose, diphosphate, and peptide tail binds at the interface of two additional monomers.

The P-loop domains-related homology group (ECOD: 2004.1) represents a large and diverse group of enzymes with ancient roots whose evolution has been discussed extensively [3-7]. P-loop domains often include two RLM motifs. Figure 6E highlights the RLM that contains the defining P-loop motif located in the RLM catalytic loop and α1 helical cap. The largest homogeneous ligand cluster is composed of the nucleotide superclass, which contains mostly ATP and GTP, whose phosphates bind to this P-loop motif. Additional diverse clusters of ligands are located near the nucleotide cluster. This ligand diversity likely stems from the origins of P-loops in early metabolism and the successful adaptation of these enzymes to the diversification of life [7].

Another functionally diverse superfamily of RLM enzymes is the HUP domain homology group (ECOD: 2005.1). HUP domain ligands are located between RLM β1, catalytic loop, α1 and β3 and the following loop (Fig 6F). The prevalent cluster in this case belongs to the nucleotide superclass, which is mostly represented by ATP and its derivatives, whose binding and evolution is discussed in detail in the following section describing the divergent evolution of nucleotides/nucleosides binding sites. As observed in the distribution of ligand clusters (Fig 6F, colored spheres), HUP domains catalyze many reactions using diverse ligand types whose functional classification and diversification have been discussed elsewhere [8, 9].

The HAD domain-related homology group (ECOD: 2006.1) contains mostly heterogeneous clusters of ligands that interact with RLM β1, the catalytic loop, and the loops after β2 and β3 (Fig 6G). Metal cations form a small cluster (yellow spheres) between the catalytic loop and the loop after β3 and represent a common metal chelation site among HAD enzymes [10]. Diverse organic oxygen compound substrates (light orange spheres), such as alpha-D-glucose 6-phosphate, interact with loop after β2 and α-helical insertion after β1. Adaptation of HAD domain catalytic reactions (discussed in the previous section on convergence to similar functions) to a large and diverse set of ligands has been accomplished using structural features that have been outlined previously [10]. Finally, the Zn-dependent exopeptidases homology group (ECOD: 2011.1, Fig 6H) contains four major ligand clusters: organic acids and derivatives (violet spheres), organoheterocyclic compounds (green), metal cations (mostly Zn, yellow), and benzenoids (brown). These clusters interact with the catalytic loop, crossover loop, loop after β3, and some of the C-terminal structural elements. Zn-dependent exopeptidases catalyze mainly peptidase activities using a metal cofactor: aminopeptidase (3.4.11.-), dipeptidase (3.4.13.-), and metallocarboxypeptidase (3.4.17.-), but also catalyze hydrolase activity on linear amides (3.5.1.-), linear amidines (3.5.3.9), and other compounds (3.5.99.8) as well as glutaminyl-peptide cyclotransferase activity (2.3.2.5).

**References**

1. Russell RB, Sasieni PD, Sternberg MJ. Supersites within superfolds. Binding site similarity in the absence of homology. J Mol Biol. 1998 Oct 2;282(4):903-18.
2. Laurino P, Tóth-Petróczy Á, Meana-Pañeda R, Lin W, Truhlar DG, Tawfik DS. An ancient fingerprint indicates the common ancestry of Rossmann-fold enzymes utilizing different ribose-based cofactors. PLoS Biol. 2016 Mar 3;14(3):e1002396.
3. Neuwald AF, Aravind L, Spouge JL, Koonin EV. AAA+: A class of chaperone-like ATPases associated with the assembly, operation, and disassembly of protein complexes. Genome Res. 1999 Jan 1;9(1):27-43.
4. Leipe DD, Wolf YI, Koonin EV, Aravind L. Classification and evolution of P-loop GTPases and related ATPases. J Mol Biol. 2002 Mar 15;317(1):41-72.
5. Iyer LM, Leipe DD, Koonin EV, Aravind L. Evolutionary history and higher order classification of AAA+ ATPases. J Struct Biol. 2004 Apr 1;146(1-2):11-31.
6. Ma BG, Chen L, Ji HF, Chen ZH, Yang FR, Wang L, et al. Characters of very ancient proteins. Biochem Biophys Res Commun. 2008 Feb 15;366(3):607-11.
7. Caetano-Anollés G, Wang M, Caetano-Anollés D, Mittenthal JE. The origin, evolution and structure of the protein world. Biochem J. 2009 Feb 1;417(3):621-37.
8. Aravind L, Anantharaman V, Koonin EV. Monophyly of class I aminoacyl tRNA synthetase, USPA, ETFP, photolyase, and PP‐ATPase nucleotide‐binding domains: implications for protein evolution in the RNA world. Proteins. 2002 Jul 1;48(1):1-4.
9. Dessailly BH, Redfern OC, Cuff AL, Orengo CA. Detailed analysis of function divergence in a large and diverse domain superfamily: toward a refined protocol of function classification. Structure. 2010 Nov 10;18(11):1522-35.
10. Burroughs AM, Allen KN, Dunaway-Mariano D, Aravind L. Evolutionary genomics of the HAD superfamily: understanding the structural adaptations and catalytic diversity in a superfamily of phosphoesterases and allied enzymes. Journal of molecular biology. 2006 Sep 1;361(5):1003-34.
