# Supplementary material for: Functional analysis of Rossmann-like domains reveals convergent evolution of topology and reaction pathways
Source: PLoS Comput Biol. 2019 Dec 23;15(12):e1007569. doi: 10.1371/journal.pcbi.1007569 (PMC6957218; doi:10.1371/journal.pcbi.1007569)
Supplement: S4 Appendix — (DOCX) [file pcbi.1007569.s004.docx]

**Description of 5’-deoxyribonucleosides ligands class binding modes for major H-groups.**

Different homology groups reveal alternate binding modes such important compounds as SAM and SAH. Fig 7B shows SAH bound to the Rossmann-related nicotinamide N-methyltransferase (EC: 2.1.1.1). The SAH adenine ring interacts with crossover residues using a binding mode similar to the adenine component of NAD in NAD(P)-binding Rossmann-fold domain homologs from this group (e.g. e1sc6A2). The ribose ring binds to a G-rich motif in the RLM catalytic loop and last residue of β2. Homocysteine interacts with α1 of RLM, replacing the NAD diphosphate in NAD(P)-binding Rossmann-folds. This SAH binding mode is conserved throughout the Rossmann-related homology group.

A different SAH binding mode (Fig 7C) is used by alpha/beta knot methyltransferases (ECOD: 2488.1). Interestingly, the knot substructure in this homology group forms a unique second RLM C-terminal to the canonical motif. SAH binds to C-terminal RLM knot, with the plane of the adenine ring parallel to the β-sheet and interacting with residues from the RLM catalytic loop and C-terminal of β3. The ribose ring interacts with the last residue of β1 and the crossover loop. The curved homocysteine points towards the loop C-terminal to the β3. These methyltransferases described above exemplify convergent functional evolution, where different folds with different binding modes of the same ligand provide similar function. One alpha/beta-knot domain, SAM-dependent rRNA:acp-transferase Tsr3, has slightly a different binding mode of this compound’s class (PDB: 5APG). Tsr3 binds Se-adenosyl-L-selenomethionine as well as the C-terminal RLM knot with the same location of adenine and ribose rings.

However, methyltransferases are not the only enzymes that bind 5’-deoxyribonucleosides. Fig 7D shows MTA/AdoHcy nucleosidase (EC: 3.2.2.9) from the purine and uridine phosphorylases H-group (ECOD: 2011.3) bound to 5'-deoxy-5'-methylthioadenosine (MTA) as a substrate. This enzyme possesses a unique binding mode of MTA (as compared to the chemically similar SAH ligands) which is likely determined by the twist and extension of the β3 of RLM (red in Fig 7D). The adenine ring plane adopts a 45º angle relative to the plane of the middle β-sheet and interacts with the extended portion of the RLM β3 and additional elements C-terminal to the RLM. The ribose ring interacts with residues from the RLM catalytic and crossover loops, and residues C-terminal to the RLM. The fourth binding mode for 5’-deoxyribonucleosides (Fig 7E) is revealed by a relatively small homology group of bacterial fluorinating enzyme C-terminal domains (ECOD: 2111.37). A representative enzyme, 5’-fluoro-5’-deoxyadenosine synthase (EC: 2.5.1.63) catalyze the formation of a C–F bond by combining SAM and F^-^ to generate 5’-fluoro-5’-deoxyadenosine and L-methionine [1]. The adenine ring plane is parallel to the β-sheet of the domain and interacts with RLM crossover loop. The homocysteine carboxyl component is in the perpendicular plane relative to the middle β-sheet, interacting with the crossover loop and α1.

**References**

1. Dong C, Huang F, Deng H, Schaffrath C, Spencer JB, O'hagan D, et al. Crystal structure and mechanism of a bacterial fluorinating enzyme. Nature. 2004 Feb;427(6974):561.
