# Supplementary figures and images for: Functional analysis of Rossmann-like domains reveals convergent evolution of topology and reaction pathways
Source: PLoS Comput Biol. 2019 Dec 23;15(12):e1007569. doi: 10.1371/journal.pcbi.1007569 (PMC6957218; doi:10.1371/journal.pcbi.1007569)

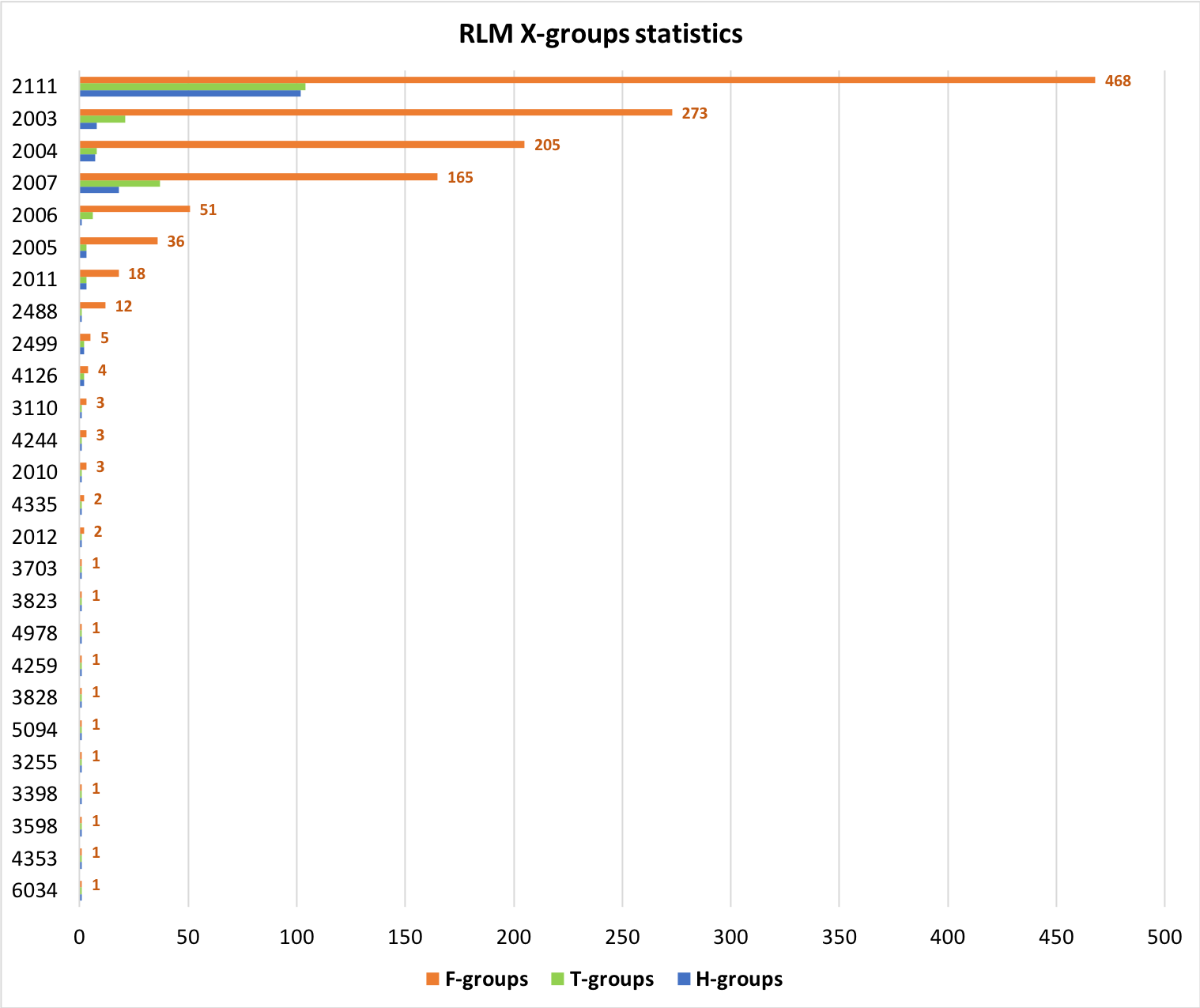

Supplement: S1 Fig — (PNG) [file pcbi.1007569.s005.png]

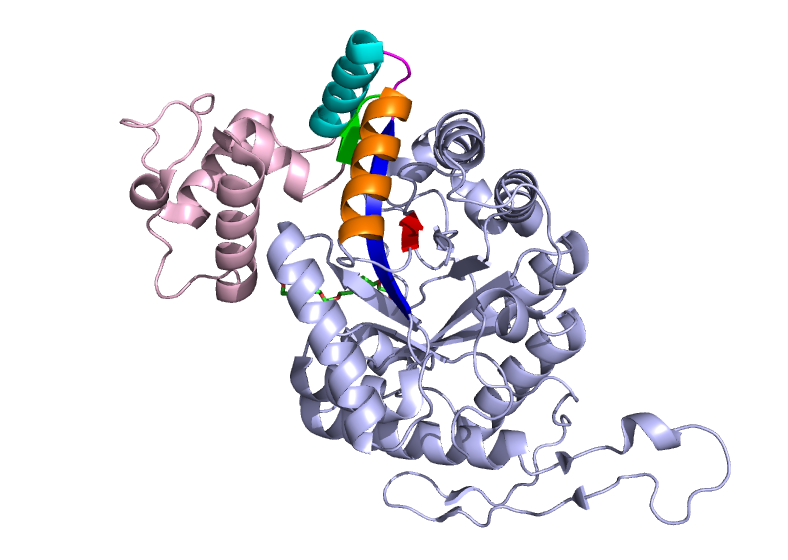

Supplement: S2 Fig — Polyethylene glycol is represented by sticks and colored by element. Moving part is colored in light pink. (PNG) [file pcbi.1007569.s006.png]

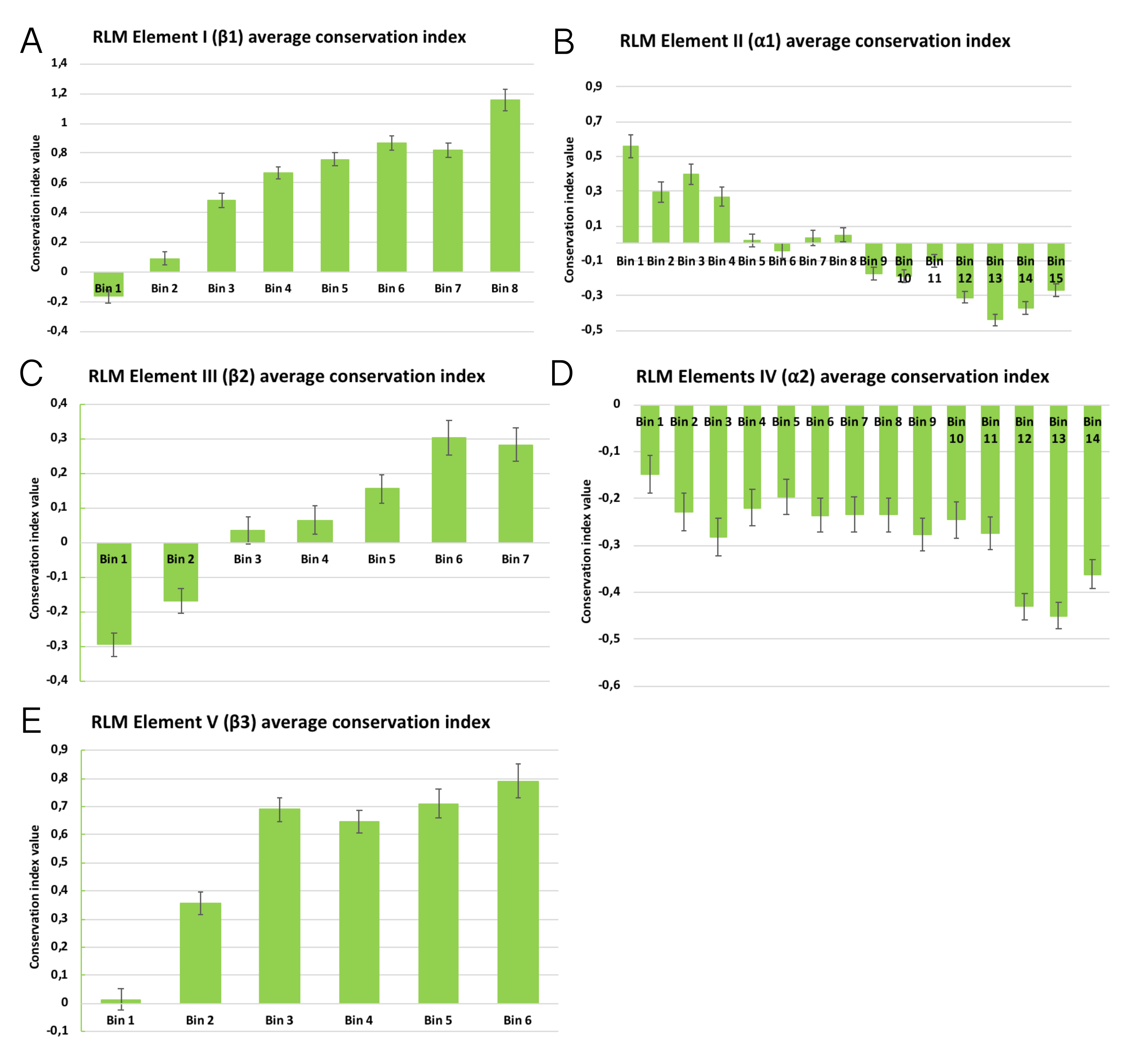

Supplement: S3 Fig — (PNG) [file pcbi.1007569.s007.png]

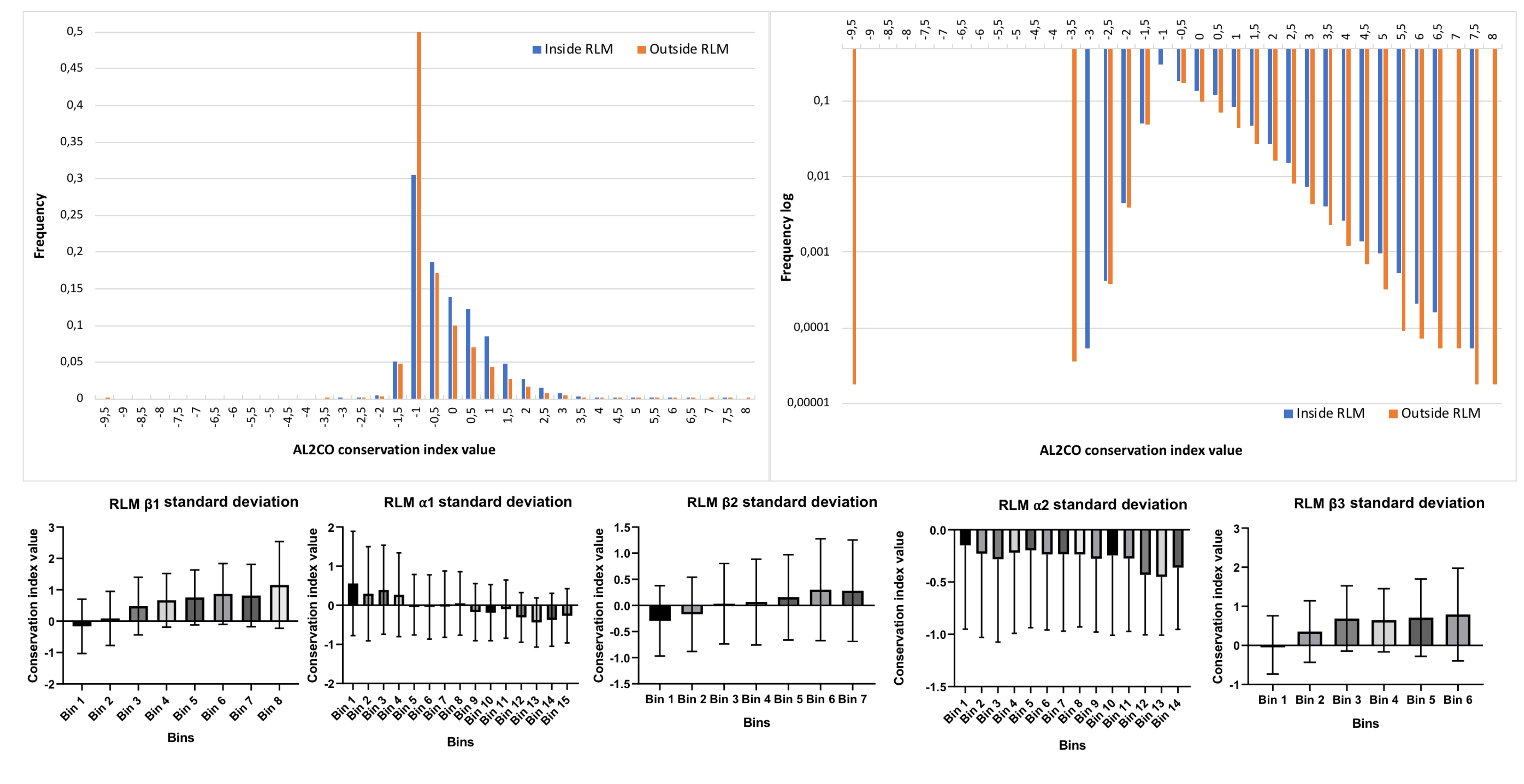

Supplement: S4 Fig — (A) Normal scale. (B) Logarithmic scale. (C-G) Length distribution of all RLM elements among representative domains. (PNG) [file pcbi.1007569.s008.png]

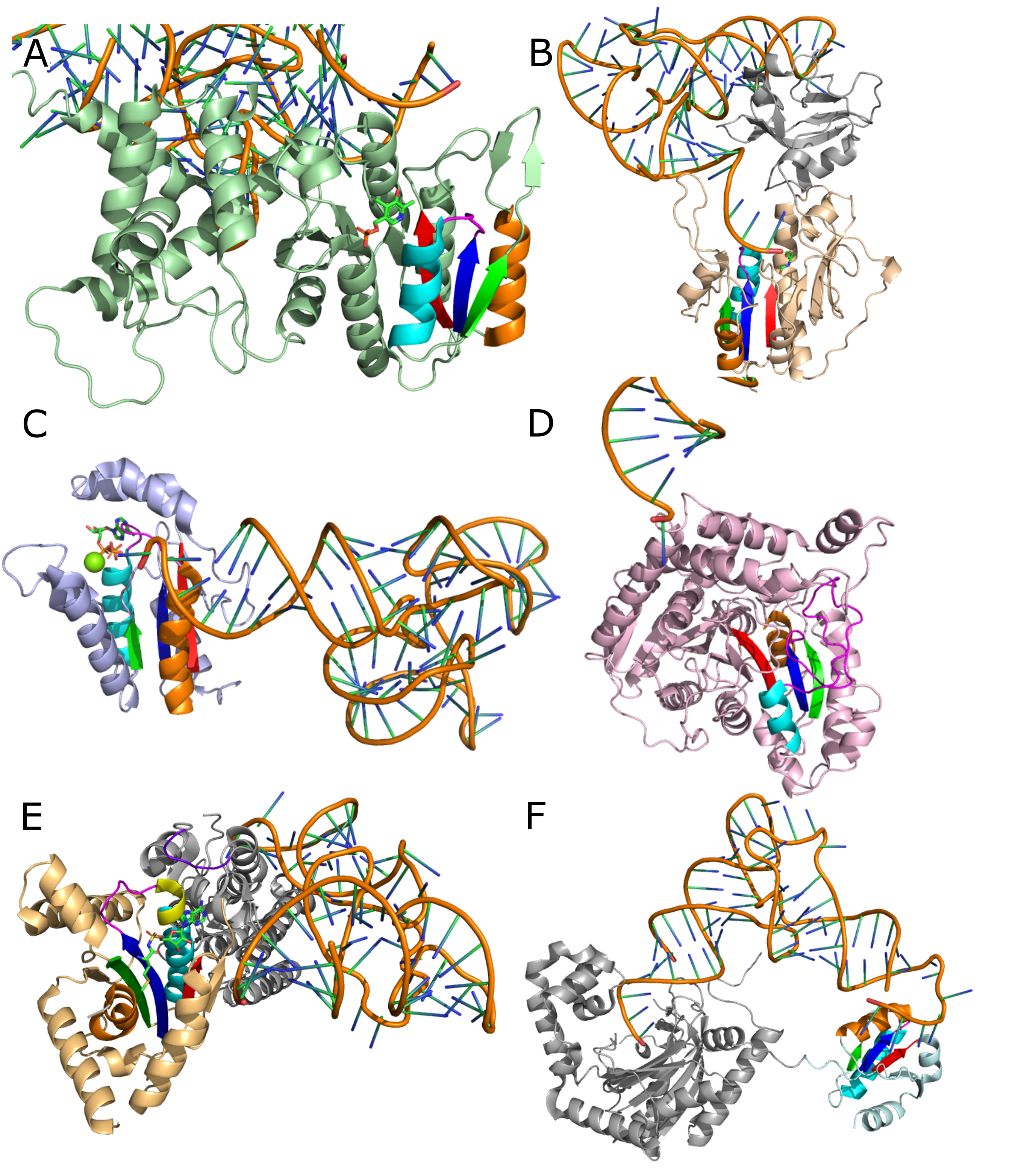

Supplement: S5 Fig — (A) PLP-dependent transferase (EC: 2.9.1.2, ECOD: e4zdoA1) binds tRNA and PLP (shown as sticks, colored by element). (B) Formyltransferase (EC: 2.1.2.9, ECOD: e2fmtA2) binds tRNA and N-formylmethionine (shown as sticks, colored by element). (C) O-Phosphoseryl-tRNA kinase (EC: 2.7.1.164, ECOD: e3am1A1) binds tRNA, ATP (shown as sticks, colored by element) and Mg (green sphere). (D) Glutaminyl-tRNA synthase RLM domain does not interact with tRNA. (E) Methionyl-tRNA synthetase class I (EC: 6.1.1.10, ECOD: e2ct8A2) binds tRNA and 5'-O-[(L-Methionyl)-sulphamoyl]adenosine (shown as sticks, colored by element). HIGH motif colored in yellow, KMSKS motif colored in purple. (F) Histidinyl-tRNA synthetase (EC: 6.1.1.21, ECOD: e4rdxA2) binds tRNA. (A-F) RLM colored in rainbow. (PNG) [file pcbi.1007569.s009.png]

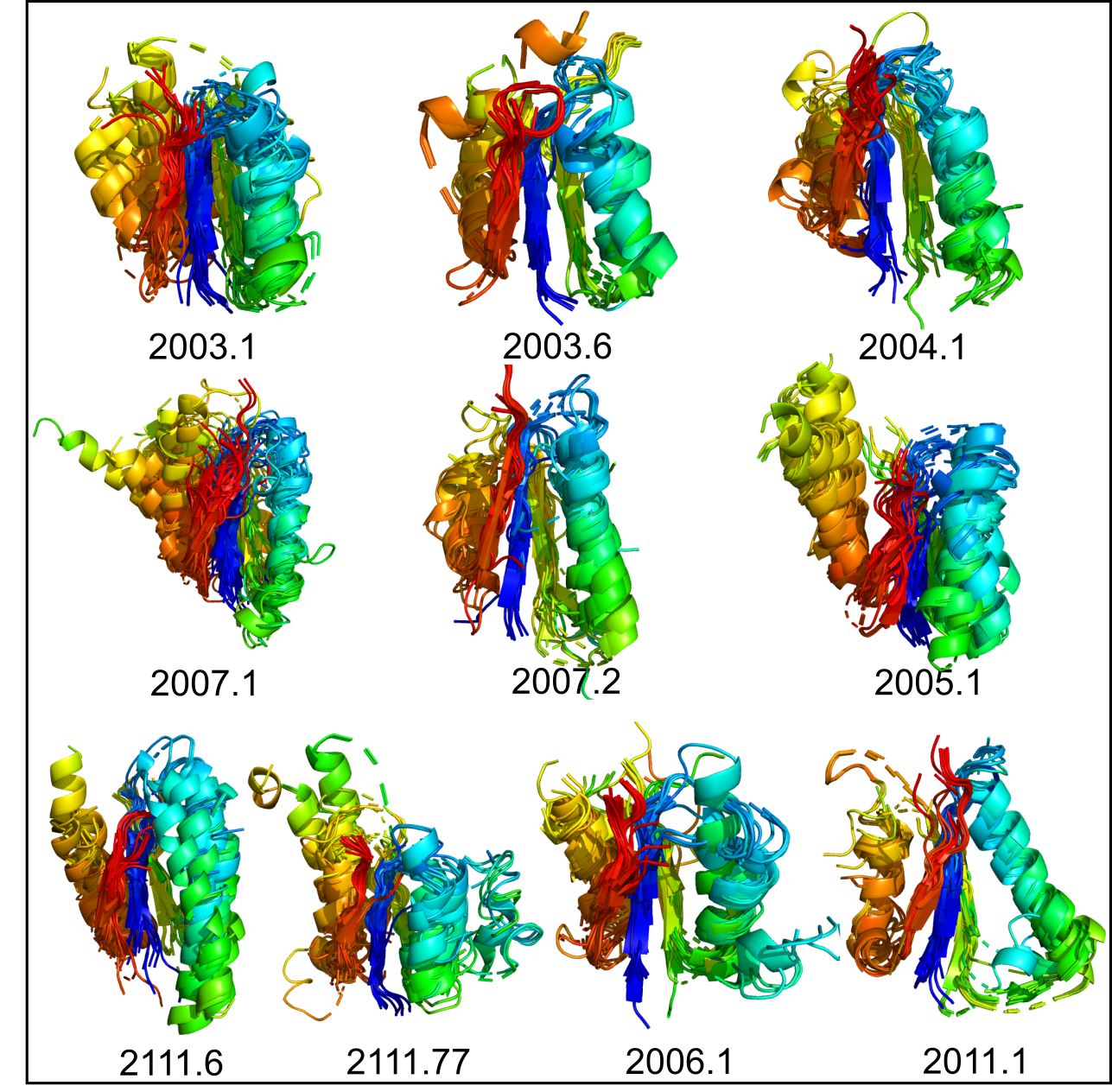

Supplement: S6 Fig — (PNG) [file pcbi.1007569.s010.png]

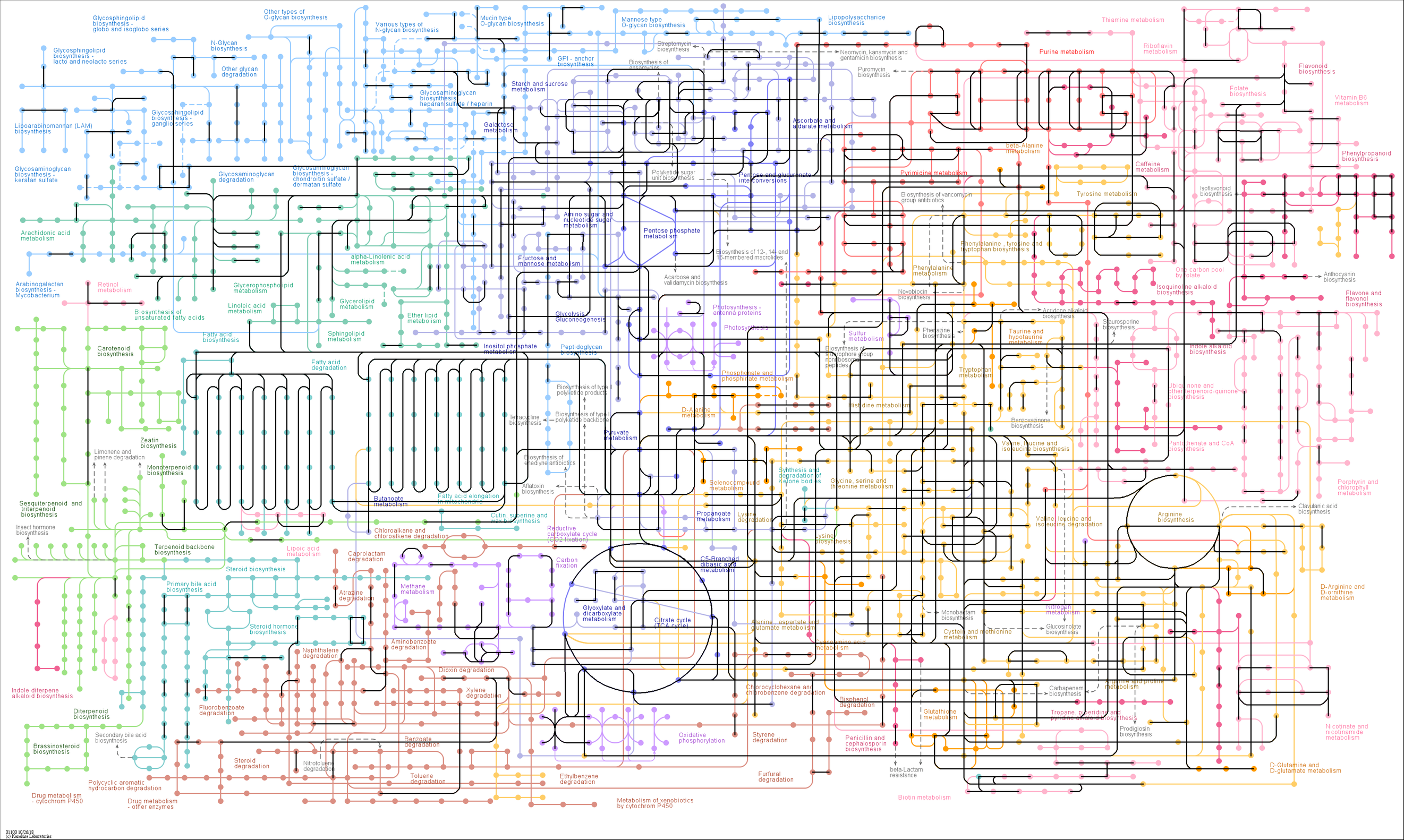

Supplement: S7 Fig — RLM protein EC numbers (black arrows) mapped to KEGG reference metabolic pathways function in all major categories: glycam biosynthesis and metabolism (light blue), lipid metabolism (green), metabolism of terpines and polyketides (lime green), xenobiotics biodegredation and metabolism (salmon), carbohydrate metabolism (blue), amino acid metabolism (orange), energy metabolism (purple), nucleotide metabolism (red), metabolism of cofactors and vitamins (pink), metabolism of other amino acids (dark orange), and biosynthesis of other secondary metabolites (magenta). (PNG) [file pcbi.1007569.s011.png]

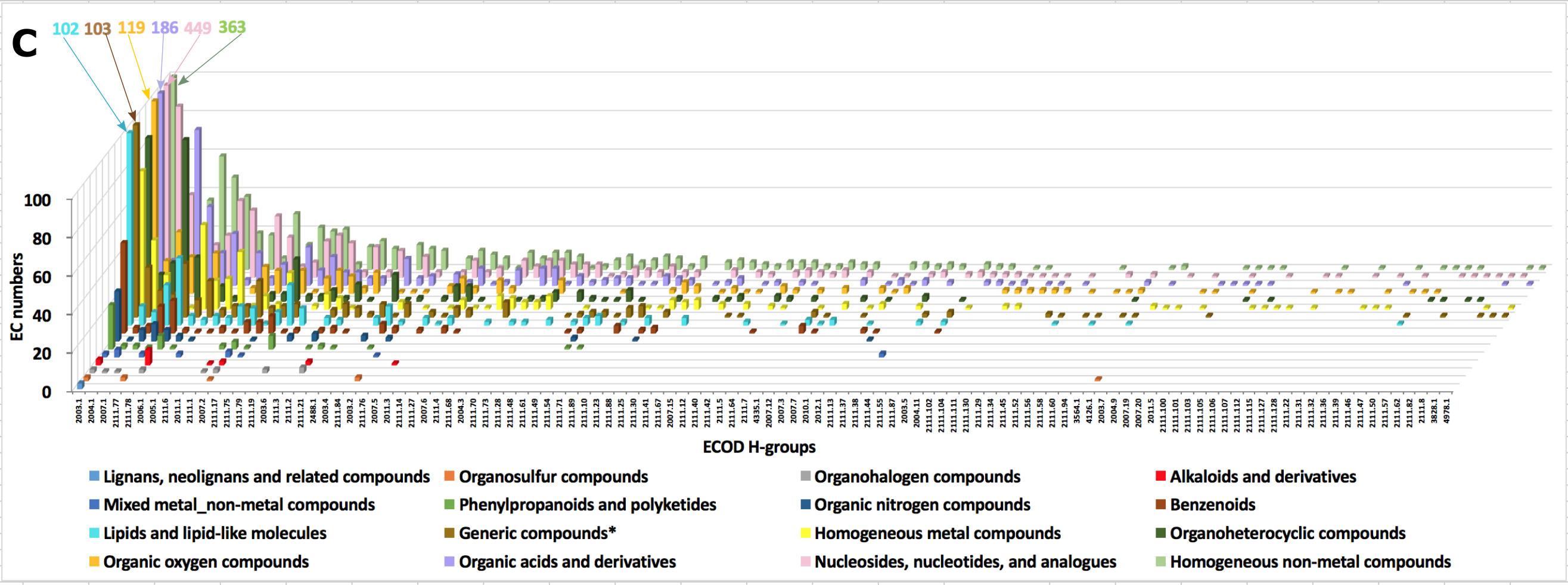

Supplement: S8 Fig — Combined KEGG compounds and UniProt cofactors (colored and classified according to ClassyFire superclass in legend) by assigned EC reaction count (Y-axis, cutoff at 100, with total number for EC>100 indicated) are distributed across ECOD Homology groups (X-axis). (PNG) [file pcbi.1007569.s012.png]
