# Supplementary material for: Functional analysis of Rossmann-like domains reveals convergent evolution of topology and reaction pathways
Source: PLoS Comput Biol. 2019 Dec 23;15(12):e1007569. doi: 10.1371/journal.pcbi.1007569 (PMC6957218; doi:10.1371/journal.pcbi.1007569)
Supplement: S3 Table — *Classification of some of ECOD X-groups changed since time of initial paper submission. (DOCX) [file pcbi.1007569.s015.docx]

| **ECOD X-groups in “α/β three-layered sandwiches” A-group*** | **RLM / non RLM** |
| --- | --- |
| Rossmann-like | RLM |
| C-terminal domain in CAC2185-like proteins | RLM |
| P-loop domains-like | RLM |
| EreA/ChaN-like | RLM |
| HUP domain-like | RLM |
| HAD domain-like | RLM |
| Flavodoxin-like | RLM |
| Nucleic acid-binding domain of nonstructural protein 3 | non RLM |
| EDD domain | RLM |
| Phosphorylase/hydrolase-like | RLM |
| LigB-like | RLM |
| Other Rossmann-like structures with the crossover | RLM |
| N-terminal domain in NTD biosynthesis operon protein NtdA | non RLM |
| Flavivirus non-structural protein 1 (NS1) a/b domain | RLM |
| STT3/PglB/AglB core domain | RLM |
| C-terminal domain in arabinosyltransferase C | non RLM |
| Nqo1 FMN-binding domain-like | RLM |
| beta-carbonic anhydrase-like | RLM |
| a/b domain in QueA-like proteins (Pfam 02547) | non RLM |
| a/b domain in AF0625-like proteins | RLM |
| CbiG linker domain | non RLM |
| CobE/CbiG C-terminal domain-like | non RLM |
| a/b domain in CV3147-like proteins | non RLM |
| alpha/beta knot | RLM |
| Peptidyl-tRNA hydrolase II | non RLM |
| Subtilisin-like | RLM |
| Insert domain in hypothetical protein PF0380 | non RLM |
| a/b domain in flagellar biosynthesis protein flhA | RLM |
| a/b domain in family 98 glycoside hydrolases | RLM |
| UPF0302 protein BA_1542/GBAA1542/BAS1430 | non RLM |
| Cellulose Synthase Subunit B a/b domain | RLM |
| Core protein P7 | RLM |
| Type III R-M system modification subunit C-terminal domain | non RLM |
| Restriction endonuclease-like | non RLM |
| ClpP/crotonase | non RLM |
| The "swivelling" beta/beta/alpha domains | non RLM |
| Barstar (barnase inhibitor) | non RLM |
| Ribosomal protein L13/L15p/L18e/L32e | non RLM |
| C-terminal subdomain in Lon-related proteases catalytic domains | non RLM |
| Atg7 N-terminal domain-like | non RLM |
| MurF and HprK N-domain-like | non RLM |
| DTD-like | non RLM |
| Cell-division inhibitor MinC, N-terminal domain | non RLM |
| CRISPR-associated Csx3 | non RLM |
| SpoIIaa-like | non RLM |
| Small protrusion domain in cytoplasmic polyhedrosis virus capsid shell protein | non RLM |
| Polycomb protein Eed insertion domain | non RLM |
| HSP90 C-terminal a/b domain | RLM |
| STING C-terminal domain | RLM |
| C-terminal domain of poxin | non RLM |
| OmpH-like | RLM |
| STIV B116-like | RLM |
